# A Factorial Randomized Controlled Trial to Optimize User Engagement With a Chatbot-Led Parenting Intervention: Protocol for the ParentText Optimisation Trial

**DOI:** 10.2196/52145

**Published:** 2024-05-03

**Authors:** Maria Da Graca Ambrosio, Jamie M Lachman, Paula Zinzer, Hlengiwe Gwebu, Seema Vyas, Inge Vallance, Francisco Calderon, Frances Gardner, Laurie Markle, David Stern, Chiara Facciola, Anne Schley, Nompumelelo Danisa, Kanyisile Brukwe, GJ Melendez-Torres

**Affiliations:** 1 University of Oxford Oxford United Kingdom; 2 Parenting for Lifelong Health Oxford United Kingdom; 3 University of Cape Town Cape Town South Africa; 4 University of Fort Hare Eastern Cape South Africa; 5 Innovations in Development, Education and the Mathematical Sciences International Reading United Kingdom; 6 mothers2mothers Mpumalanga South Africa; 7 Clowns Without Borders Cape Town South Africa; 8 University of Exeter Exeter United Kingdom

**Keywords:** parenting intervention, chatbot-led public health intervention, engagement, implementation science, mobile phone

## Abstract

**Background:**

Violence against children (VAC) is a serious public health concern with long-lasting adverse effects. Evidence-based parenting programs are one effective means to prevent VAC; however, these interventions are not scalable in their typical in-person group format, especially in low- and middle-income countries where the need is greatest. While digital delivery, including via chatbots, offers a scalable and cost-effective means to scale up parenting programs within these settings, it is crucial to understand the key pillars of user engagement to ensure their effective implementation.

**Objective:**

This study aims to investigate the most effective and cost-effective combination of external components to optimize user engagement with ParentText, an open-source chatbot-led parenting intervention to prevent VAC in Mpumalanga, South Africa.

**Methods:**

This study will use a mixed methods design incorporating a 2 × 2 factorial cluster-randomized controlled trial and qualitative interviews. Parents of adolescent girls (32 clusters, 120 participants [60 parents and 60 girls aged 10 to 17 years] per cluster; N=3840 total participants) will be recruited from the Ehlanzeni and Nkangala districts of Mpumalanga. Clusters will be randomly assigned to receive 1 of the 4 engagement packages that include ParentText alone or combined with in-person sessions and a facilitated WhatsApp support group. Quantitative data collected will include pretest-posttest parent- and adolescent-reported surveys, facilitator-reported implementation data, and digitally tracked engagement data. Qualitative data will be collected from parents and facilitators through in-person or over-the-phone individual semistructured interviews and used to expand the interpretation and understanding of the quantitative findings.

**Results:**

Recruitment and data collection started in August 2023 and were finalized in November 2023. The total number of participants enrolled in the study is 1009, with 744 caregivers having completed onboarding to the chatbot-led intervention. Female participants represent 92.96% (938/1009) of the sample population, whereas male participants represent 7.03% (71/1009). The average participant age is 43 (SD 9) years.

**Conclusions:**

The ParentText Optimisation Trial is the first study to rigorously test engagement with a chatbot-led parenting intervention in a low- or middle-income country. The results of this study will inform the final selection of external delivery components to support engagement with ParentText in preparation for further evaluation in a randomized controlled trial in 2024.

**Trial Registration:**

Open Science Framework (OSF); https://doi.org/10.17605/OSF.IO/WFXNE

**International Registered Report Identifier (IRRID):**

DERR1-10.2196/52145

## Introduction

### Background and Rationale

The detrimental consequences of physical and emotional violence against children (VAC) have been associated with multiple lifelong health disorders [1-3]. VAC negatively affects children’s physical (ie, atypical structural development of the brain and altered functioning of the endocrine system) and cognitive (ie, harsh corporal punishment in children increases the risk of alcohol and substance abuse in adulthood and affects cognitive processes such as reasoning and attention) development [1,4]. Children exposed to violence also have an increased risk of experiencing and perpetrating domestic violence in adulthood [5]. VAC has also been associated with inequalities in academic achievements for boys and girls, with children exposed to physical and sexual violence being 13% less likely to complete high school [6].

While VAC is a global public health concern, rates are higher in low- and middle-income countries (LMICs) such as South Africa [7]. In a study with 3515 South African children, the prevalence of lifetime physical and emotional abuse was 56% and 35%, respectively [8,9]. Child sexual abuse is also widespread in the country, with a nationally representative cross-sectional survey reporting that 14.6% of girls and 10% of boys had experienced this form of abuse [10]. The economic costs of VAC in South Africa are significant. In the fiscal year 2015-2016, an estimated 4.3% of the country’s gross domestic product was lost to VAC [11]. In addition, the expenditure on childcare and protection attributable to VAC was estimated to be 1.6 billion South African rands (US $124 million) [11].

Inequitable gender norms, such as societal acceptance of male aggression toward women and children, further impede adolescent girls’ healthy development and education in South Africa [12]. In addition, adolescent girls are a recognized vulnerable group and susceptible to increased risk of sexually transmitted infections, including HIV, and violence, risks that stem from sexist social principles (ie, adolescent girls tend to be sexualized because of their physical development changes) and structural constraints (eg, poverty, socioeconomic inequality, and high levels of alcohol abuse and crime within communities) [12,13]. In 2018, approximately 16% of adolescent girls aged 15 to 17 years reported that they had experienced sexual abuse perpetrated mainly by a known male adult [10]. In addition, 21% of girls aged 15 to 21 years indicated that their first sexual experience was either coerced or forced [14].

While legislative development efforts have been made to address VAC in South Africa, entrenched social norms condoning physical and emotional abuse toward children are hard to change [15]. Research has demonstrated that a healthy growing environment positively affects children’s early brain development, providing the foundations for developing long-term cognitive abilities such as language and reading [4]. Previous research has linked parental engagement in early childhood with better language development and literacy outcomes in children [16,17]. Open access, evidence-based parenting interventions such as the Parenting for Lifelong Health (PLH) suite of programs offer a prime opportunity to address such norms both within the family and at the broader societal level and create a safer environment for children. These interventions, typically delivered as in-person, group-based programs, have been effectively implemented worldwide to increase responsive and positive parenting [18]. However, in low-resource settings, factors such as employment, transportation, and childcare costs represent a significant financial and accessibility barrier to in-person parenting programs for many participants [19-21].

### Prospects for Implementing Chatbot-Led Public Health Interventions

In recent years, digital interventions have been used as a delivery mechanism to address the structural barriers to accessing in-person parenting programs [22,23]. For example, PLH has recently developed digital adaptations of their in-person programs in response to restrictions placed on in-person parenting programs during the COVID-19 pandemic and challenges reported by implementing partners in delivering programs at scale in low-resource settings in sub-Saharan Africa. One such PLH adaptation is ParentText, an internet-based self-guided chatbot sent via instant messaging platforms such as WhatsApp and Telegram [24]. Text-based chatbots delivered through instant messaging platforms are being widely explored in the digitalization of public health programs, including parenting interventions. The Oxford Dictionary defines a chatbot as “a computer programme that can hold a conversation with a person, usually over the internet” [25]. They are also referred to as conversational software applications designed to interact in humanlike in-text or text-to-speech conversations with human users [26]. There is evidence suggesting that chatbots have been successfully applied to support health promotion interventions focused on improving mental well-being [27], reducing alcohol consumption [28], and supporting weight loss [29,30].

In 2022, according to World Bank estimates, 70% of South Africa’s population had internet access, and there were 162 mobile phone subscriptions per 100 people [31]. This level of digital coverage makes internet-based interventions such as chatbots a promising delivery channel for parenting programs in the country. Several chatbot-led public health interventions have already been successfully implemented in South Africa. MomConnect, a chatbot that provides pre- and postnatal health information to pregnant women and new mothers to improve their health and that of their babies, has been operating in the country since 2014. By 2016, the chatbot, an initiative of the South African National Department of Health, had reached over half a million users [32]. In 2020, HealthConnect was launched to provide COVID-19–related health information to the general population, and it was estimated that the platform had reached tens of millions of users [33]. Another successful chatbot-led intervention is ChattyCuz, a gamified interactive chatbot targeted at young women to support them in maintaining healthy dating relationships and preventing intimate partner violence (IPV). In a randomized controlled trial with 19,643 young women, researchers reported 59% retention in the intervention [34]. Participants reported improved gender beliefs and reduced rates of IPV because of their interaction with ChattyCuz [34].

Despite the promise of chatbot interventions, the engagement rate of users enrolled in digital parenting interventions and public health interventions more broadly remains low [35-37]. Several factors that affect human-chatbot interaction, such as user trust, satisfaction, and external structures (eg, difficult access to smartphones and the internet and low levels of digital literacy), may undermine their effective implementation [38]. To date, research has primarily focused on investigating the effectiveness of chatbot-led public health interventions on changing behavior and health outcomes [38,39]. However, few digital public health interventions have investigated the factors underlying participant engagement [38,39]. Engagement can be conceptualized as “the extent (e.g., duration of interaction, frequency of contact and depth or variety of content used) of usage” and as “a subjective experience characterised by attention, interest and affect” [40]. Previous research has suggested 4 distinguishable attributes inherent to engagement: point of engagement (ie, initiation stage), period of sustained engagement (ie, maintenance stage), disengagement (ie, period of interruption), and re-engagement [41]. Even though understanding how each of these stages occurs is of extreme relevance to designing the most optimal digital health interventions, this study will focus on the second stage. The rationale behind this decision stems from previous research reporting higher dropout rates after 2 weeks of enrollment in digital health interventions [29,42,43]. In addition, there is a paucity of research focused on investigating how users remain engaged with parenting chatbots [44].

High engagement rates are presented in the literature as an average open-app ratio of 17.71 and 12.14 times over a 2-week period [29,42]. Another complication is that high rates of participant dropout challenge this research, and the determinants of attrition are uncertain as previous studies have lacked statistical power to analyze engagement [27,38]. Another critical factor in user dropout in human-chatbot interaction is the interaction breakdown due to the chatbot’s inability to understand users’ input, which may arouse negative emotional responses in users [45-47].

Cultural determinants are another potential factor affecting user enrollment and engagement with digital public health interventions. Cultural determinants of health refer to factors related to perceptions, relationships, and cultural identity that interact with society and influence an individual’s good health and well-being [48]. Once identified, efforts can be made to promote positive cultural aspects supporting user engagement with chatbot-led public health interventions. In a Kenyan study, intent to use mobile health (mHealth) was associated with the degree to which individuals perceived that their peers had a favorable opinion about their use of mHealth [49]. In Pakistan, participants’ perceptions of how easy it was to use an mHealth intervention and their positive attitude toward digital health interventions were associated with their intent to use them [50]. This finding highlights the need for increased user participation in the design process of digitally supported interventions [51] and competent cross-cultural delivery of digital parenting programs. There is also a need to strengthen the evidence base to optimize chatbot-led parenting interventions in LMICs. Such research should (1) assess the financial and implementation constraints to institutionalize them across stakeholders efficiently, (2) develop a culturally sensitive approach in their implementation to ensure effectiveness in terms of the acceptability and satisfaction of users, and (3) evaluate their effectual impact in increasing positive parenting behavior and reducing VAC.

### Objectives

#### Overview

The ParentText Optimisation Trial aims to optimize user engagement through a chatbot-led parenting intervention delivered to parents (ie, any adult responsible for the care and well-being of a child regardless of biological relationship) and their adolescent girls aged 10 to 17 years in South Africa. It will test the relative effectiveness and cost-effectiveness of two intervention components designed to support user engagement with the ParentText chatbot: (1) remotely facilitated WhatsApp support groups delivered to participating parents and (2) in-person sessions delivered to parents and their adolescent girls. In addition, this study will investigate the effectiveness of selected intervention component levels on adolescent and parent behavioral, mental health, and educational outcomes.

#### Primary Objectives

The primary objectives are as follows:

1. To examine the effectiveness and cost-effectiveness of selected component levels on the primary engagement outcome of the total number of modules completed in ParentText.
2. To examine the effectiveness and cost-effectiveness of selected component levels on secondary engagement outcomes in terms of the percentage of modules and goals completed, the rate of self-reported completion of home practice activities based on the self-reported intention to do home practice activities, and the total number of overall interactions of participants with the chatbot.

#### Secondary Objectives

The secondary objectives are as follows:

1. To examine the effectiveness and cost-effectiveness of intervention component levels on parent and adolescent behavioral, mental health, and educational outcomes assessed at baseline and 6 weeks after the baseline.
2. To identify the cultural determinants (perceived ease of use, perceived usefulness, hedonic motivation, habit, price value, and social influence) that significantly contribute to improved behavioral intentions of parents to engage with ParentText.
3. To identify how behavioral intention significantly contributes to improved primary and secondary engagement outcomes.
4. To identify participants’ baseline characteristics and cultural determinants that predict primary and secondary engagement outcomes of parents with ParentText.
5. To explore the role of baseline characteristics as potential moderators of the effectiveness of the intervention components on primary and secondary engagement outcomes.
6. To identify participants’ characteristics that moderate the effect of behavioral intention on primary and secondary engagement outcomes.
7. To explore the role of baseline characteristics as potential moderators of the effectiveness and cost-effectiveness of intervention components on parent and adolescent behavioral, mental health, and educational outcomes assessed at baseline and 6 weeks after the baseline.
8. To qualitatively explore parents’ perceptions of challenges in engaging with ParentText and their perception of the most feasible and acceptable components and component levels for its implementation in South Africa.
9. To identify the incremental cost of adding each of the component levels in the ParentText implementation package.
10. To select the most effective and cost-effective combination of components and component levels to be tested further in a randomized controlled trial in 2024.

### Trial Design

#### Overview

This study design was derived from the multiphase optimization strategy framework developed by Collins [52], which consists of 3 stages: preparation, optimization, and evaluation. In the multiphase optimization strategy, intervention optimization is the process of evaluating and identifying from a set of intervention components the combination with the most effective and scalable design that can be obtained subject to existing constraints such as limited financial and human resources [49]. The preparation phase of this study took place between October 2022 and February 2023. It comprised a formative evaluation with stakeholders from government and nongovernmental organizations (NGOs) in South Africa. The findings of this phase informed the selection of the intervention components for inclusion in this trial.

The ParentText Optimisation Trial will be conducted from August 21, 2023, to November 10, 2023, in Mpumalanga, South Africa. It will use a 2 × 2 cluster-randomized experimental design to investigate the effectiveness and cost-effectiveness of two components delivered externally from the ParentText chatbot: (1) in-person sessions with parents and adolescent girls (1 or 4 sessions) and (2) facilitated WhatsApp support groups with parents (yes or no). All caregivers and adolescent girls will be onboarded to ParentText in the in-person session (the first session for those assigned to receive 4 in-person sessions). The 2 × 2 factorial trial will include four experimental conditions (Table 1):

1. Condition 1: ParentText chatbot and 1 in-person session (onboarding to ParentText) with parents and adolescent girls
2. Condition 2: ParentText chatbot, 1 in-person session (onboarding to ParentText) with parents and adolescent girls, and facilitated WhatsApp groups for parents
3. Condition 3: ParentText chatbot and 4 in-person sessions (with the first session focused on onboarding to ParentText) with parents and adolescent girls
4. Condition 4: ParentText chatbot, 4 in-person sessions (with the first session focused on onboarding to ParentText) with parents and adolescent girls, and facilitated WhatsApp groups for parents

The study will randomly assign 32 clusters split equally (n=8 clusters) across the 4 different treatment conditions. A total of 1920 parents and their adolescent girls aged 10 to 17 years (approximately 60 parents and 60 adolescent girls per cluster) will be recruited to participate in the study. Each cluster will be randomly assigned a dedicated community-based facilitator to deliver the in-person and WhatsApp support group sessions. The study will adopt a between-cluster assignment to prevent contamination across experimental conditions.

**Table 1:** Experimental conditions for a 2 × 2 factorial trial (n=32 clusters, n=60 parent-adolescent dyads, and N=3840 participants).

Condition	Clusters^a^ (n=32), n (%)	ParentText (always on)^b^	In person (1 session vs 4 sessions)^c^	WhatsApp groups (yes vs no)^d^
1	8 (25)	On	1	No
2	8 (25)	On	1	Yes
3	8 (25)	On	4	No
4	8 (25)	On	4	Yes

^a^*Cluster* refers to parent and adolescent groups linked to a designated facilitator.

^b^*Always on* refers to the provision of the chatbot without any external support.

^c^Parents and adolescent girls will receive either 1 or 4 in-person sessions.

^d^Parents will either receive (yes) or not receive (no) facilitated WhatsApp groups.

#### Primary Hypotheses

The primary hypotheses are as follows:

1. Facilitated WhatsApp support groups (yes or no): we hypothesize that parents receiving the ParentText delivery package combined with web-based support via WhatsApp groups will have higher engagement rates than those receiving the ParentText delivery package without facilitated support via WhatsApp groups.
2. In-person sessions (1 or 4 sessions): we hypothesize that parents receiving the ParentText delivery package combined with 4 in-person sessions will have higher engagement rates than those receiving the ParentText delivery package with 1 in-person session.
3. Incremental cost-effectiveness: we hypothesize that, while the inclusion of facilitated WhatsApp groups will be more cost-effective than not including facilitated WhatsApp groups based on the incremental cost advantage, the higher dosage of in-person sessions will not be a cost-effective component.

#### Secondary Hypotheses

The secondary hypotheses are as follows:

1. Behavior outcomes: we hypothesize that the most intensive combination of component levels will have the greatest effect on parent and adolescent behavioral, mental health, and education outcomes.
2. Cultural determinants: we hypothesize that cultural determinants (perceived ease of use, perceived usefulness, hedonic motivation, habit, price value, and social influence) will be associated with more favorable intentions to engage with ParentText.
3. Behavioral intention: we hypothesize that more favorable intentions to engage with ParentText will lead to higher primary and secondary engagement rates.

The positive directionality of the effects of the 2 intervention components on engagement outcomes will be preferred in this study due to evidence of the positive impact of engagement boosters in parenting interventions [53,54]. No hypotheses will be made a priori for potential moderators of effectiveness or cost-effectiveness.

## Methods

### Study Setting

The ParentText Optimisation Trial is nested within a larger project implemented by mothers2mothers (m2m), a South African NGO, as part of their Children and Adolescents Are My Priority (CHAMP) program funded by the US Agency for International Development and the President’s Emergency Plan for AIDS Relief. The study will be conducted across 2 districts in Mpumalanga Province—Ehlanzeni (mostly rural zones) and Nkangala (rural and periurban zones)—where CHAMP was implementing the orphaned and vulnerable children program or Determined, Resilient, Empowered, AIDS-free, Mentored, and Safe (DREAMS) family-strengthening interventions to approximately 28,500 adolescent women and girls (aged between 10 and 17 years) and their caregivers in 2023. The primary home languages spoken by participants include English, isiZulu, and siSwati. The CHAMP program delivers a portfolio of age-specific, needs-based interventions aimed at reducing HIV and AIDS incidence among orphaned and vulnerable children, adolescents, and their families in communities across the province. A key component of CHAMP includes evidence-based interventions to decrease family violence and improve health and social outcomes for orphaned and vulnerable children and adolescents, such as the PLH program.

### Eligibility Criteria

Participants recruited for this trial will be parents, adolescent girls, and implementing facilitators.

Eligible clusters are communities with at least 60 households with a resident adolescent girl aged between 10 and 17 years and assigned to an m2m CHAMP implementer. Each implementer will be responsible for 60 households.

Eligible parents are those (1) aged ≥18 years and who are English, isiZulu, or siSwati speakers; (2) currently caring for an adolescent girl aged between 10 and 17 years; (3) living in the same household with the adolescent girl for a minimum of 4 nights a month over the previous 3 months; (4) having access to a mobile phone compatible with WhatsApp; (5) willing to enroll in the ParentText chatbot and receive messages via WhatsApp; and (6) who provide written informed consent to participate in the full study.

Eligible adolescents are (1) female teenagers aged between 10 and 17 years who (2) have a parent enrolled in the trial and (3) have provided written informed assent to enroll in the full study.

Eligible facilitators are (1) m2m CHAMP implementers in Mpumalanga aged ≥18 years who (2) have completed a workshop training for program delivery before the trial; (3) have a mobile phone compatible with WhatsApp and access to the internet or a data bundle; (4) speak English, isiZulu, or siSwati; and (5) have provided written informed consent to participate in the trial.

Parents with a severe learning disability or those exhibiting acute mental health disorders will be excluded due to their limited capacity to provide informed consent. Parents whose child refuses to participate in the study will not be excluded to prevent threats to internal validity. Children who have healthier relationships with their parents could be more interested in participating in the study; thus, this could lead to selection bias.

### Informed Consent

Trained m2m program facilitators will collect informed consent from adults and informed assent from adolescents before the baseline at local schools in communities where the study is taking place (Multimedia Appendices 1 and 2). Informed consent and assent forms will include clear descriptions of the intervention, study objectives, use and protection measures for participant data, and participant rights to refuse to respond to survey questions or withdraw at any point from the study. Adolescents’ participation will be conditional on their parents providing consent. Adults and adolescents who agree to participate in the intervention will be invited to sign a paper-based informed consent form to indicate their consent. Participants will also receive a hard copy of information sheets, which include contact details of the local research team and ethics board.

### Additional Consent Provisions for Collection and Use of Participant Data

Parents and adolescents will be required to have provided informed consent to participate in the CHAMP project before recruitment to the study.

### Interventions

#### Intervention Description

The core parenting intervention component will be ParentText, a chatbot that delivers personalized and gamified scheduled and on-demand messages through text, audio, and visual media based on development stages for parents of children aged 0 to 23 months, 2 to 9 years, and 10 to 17 years. Previous research has reported the acceptability and feasibility of ParentText in LMICs. A qualitative formative research study in Jamaica reported an average participant (ie, parents) engagement length of 14 days throughout the 37 days of the intervention [43]. The version of the program targeting those aged 10 to 17 years will be used in this study in alignment with the age group of adolescent girls targeted by the CHAMP program. ParentText was originally developed by the UK-based charities PLH and Innovations in Development, Education, and the Mathematical Sciences (IDEMS) International in collaboration with the United Nations Children’s Fund (UNICEF); the Universities of Oxford and Cape Town; and Clowns Without Borders South Africa (CWBSA), one of the local partners, to ensure linguistic, cultural, and contextual relevance. The main program content was derived from the PLH for Parents and Teens program, a 14-session in-person intervention developed and tested in a cluster-randomized controlled trial in South Africa [55]. Additional content was included to support adolescent mental health (UNICEF’s Helping Adolescents Thrive program [56]), adolescent educational outcomes (the LEGO Foundation’s learning through play resources [57]), and gender-based violence prevention (No Means No Worldwide). All content was translated into isiZulu and siSwati and reviewed by parenting experts who were fluent in the 2 languages.

ParentText messages are grouped into nine positive parenting goals: (1) *improve my relationship with my teen*; (2) *understand teen development*; (3) *support my teen’s education*; (4) *create structure for my teen*; (5) *improve my family’s finances*; (6) *care for my teen’s well-being*; (7) *manage my teen’s behavior*; (8) *keep my teen safe*; and (9) *have a healthy relationship with my partner*, the latter only delivered to parents who have indicated that they are in a partnered relationship. Each goal is supported by learning modules (37 in total) designed to build parent interpersonal skills through comics, videos, and text illustrating key parenting tips. ParentText also includes internal components to support user engagement, such as gamification (eg, earning badges toward goals), personalization (eg, male and female videos), and activities (eg, quizzes). Participants can select the order of goals based on their preferences after completion of the first goal (*improve my relationship with my teen*).

The goal *improve my relationship with my teen* “improve my relationship with my teen” (Table 2) will provide content on how to spend one-on-one time with the teenager and techniques to praise the adolescent and talk about feelings. For the goal “understand teen development,” parents will complete modules related to understanding teenage mental, social, and physical changes. The goal “support my teen’s education” will deliver content related to creating a fun and positive environment to support the teenager’s academic learning process. Parents will learn skills to create a routine for teenagers and establish rules for the goal “creating a structure for my teen.” The goal “improve my family’s finances” will equip parents with budgeting skills through lessons related to savings and expenses. The goal “show kindness to your teen” will deliver content related to identifying and managing stress signs and techniques for supporting adolescents. For the goal “keep my teen safe,” parents will receive content related to building skills to manage teenagers’ misbehavior and help keep their adolescents safe in the community and relationships. Finally, parents who report being in a relationship will receive additional content on how to be supportive, solve conflicts in a relationship, and share responsibilities. The number of assigned modules will vary based on the age of the child and the number of goals but will be up to a total of 37, or 32 for nonpartnered parents.

Participants will be enrolled in the intervention for 6 weeks. Participants who enroll in the hybrid format of the program will also participate in 6 weekly WhatsApp live support sessions and 3 biweekly sessions of the in-person program subject to the study components available to their cluster. Parents will participate in the intervention by interacting with ParentText and completing each of the 37 modules and 9 goals sequentially. All goals and modules will be available to parents from the moment they enroll in the parenting chatbot. The goal “Have a Healthy Relationship with my Partner” will only be available for parents who reported being in a relationship. The order in which parents choose to access the goals will not be relevant because the goals were not developed to be interdependent. Adolescents will not interact with the chatbot but will experience the program through voluntary participation in the home practice activities of their parents.

The implementation will follow a group-based format that includes 3 joint parent-and-teenager sessions on (1) emotional check-in and mindfulness-based exercises to reduce stress, (2) feedback activity on participants’ experience with ParentText, (3) core lesson supported by illustrations, (4) collaborative discussion problem-solving, and (5) practice of skills at home. Participants will be trained on skills such as family budgeting, widening circles of support, and keeping safe in the community and in relationships. WhatsApp live chat group sessions will only include text messages. The session will include (1) a welcome and check-in message sent by the m2m facilitator, (2) sharing success (parents will be encouraged to share a positive experience when trying one of the ParentText skills with their teenagers), (3) fun activity that parents will be encouraged to replicate after with their teenagers (eg, a dance move), (4) sharing challenges (challenges faced by parents when trying a new skill with their child), and (5) collaborative discussion problem-solving.

**Table 2:** Intervention modules—ParentText content.

Goal	Number of modules	Justification
Improve my relationship with my teenager	3	A stronger parent-child relationship is associated with lower levels of conflict and violence in families [58].
Understand teenager development	3	Parents who understand the development of an adolescent’s sexual and reproductive health are better equipped to create a safe and supportive environment for them. Parental monitoring, communication, and support are vital in sexual violence and VAC^a^ prevention [59].
Support my teenager’s education	5	Research suggests that parents who are involved in their adolescents’ education help provide a positive school environment by promoting the love for learning [60].
Create structure for my teenager	4	Rulemaking can decrease disputes between parents and their children. When parents set rules, they can protect their children from harmful acts or behaviors that may result in abuse by other adults [61].
Manage my teenager’s behavior	4	Managing adolescents’ behavior prevents later involvement in risky behavior, such as substance use, violence, later delinquency, and conduct problems [62].
Care for my teenager’s well-being	4	Listening actively helps adolescents feel heard, understood, less alone, and calmer. In contrast, if parents do not listen to and support their adolescents properly, this can leave them feeling defensive, frustrated, alone, or hurt [63].
Keep my teenager safe	4	Adolescents are at a higher risk of becoming victims of crime than any other age group [64].
Have a healthy relationship with my partner	5	Fatherhood programs that encourage fathers to be more engaged in caregiving and parenting show hopeful findings in terms of reducing VAC and IPV^b^ [65].
Improve my family’s finances	4	According to the family stress model, financial problems increase parents’ stress and depression, which in turn increases harsh parenting practices and child maltreatment [66].

^a^VAC: violence against children.

^b^IPV: intimate partner violence.

#### Tested Intervention Components

##### Intervention Component 1: In-Person Sessions

This trial will examine whether varying frequency of in-person human support significantly affects user engagement with the chatbot. In-person parenting programs come with the costs of training facilitators and commonly present limited community resources and infrastructure for their delivery [21]. Of the variants of human support selected for this trial, the in-person component requires a greater allocation of resources. Nevertheless, there is great value in understanding how much of the in-person component is necessary to bolster engagement in chatbot-led public health interventions. Proponents of remote support tools argue that including social networking as a tool to support participants is associated with greater engagement rates [67,68]. In supported digital interventions, participants receive technical and clinical troubleshooting assistance to bolster engagement [69]. Previous studies have reported that the frequency with which participants interacted with a counselor or clinician coach predicted their adherence to digital health interventions [67,70].

This study will compare the differential effects of delivering either 1 or 4 in-person group-based sessions to parents and their adolescents (maximum of 60 families per group). Each session will be delivered by an m2m CHAMP lead facilitator and a cofacilitator and last approximately 2 hours.

##### Intervention Component 2: Facilitated WhatsApp Support Groups

This study will also test the impact of facilitated WhatsApp support groups on user engagement in the ParentText chatbot intervention. Self-guided interventions present greater scope for scalability due to the low allocation of financial and human resources required. However, low engagement rates risk their sustainability over time. Although including engagement boosters is a common practice in parenting programs to increase parent engagement [53,54], their effectiveness in increasing engagement has been challenged in previous studies. Day and Sanders [71] found that parents benefited from phone support when completing the Triple P Online self-guided parenting program. Parents in the practitioner-supported condition engaged more with the program, completing more modules and reporting greater program satisfaction [71]. However, Epstein et al [72] found that providing opportunities for parents to interact via Facebook groups while participating in a self-directed family intervention did not increase engagement or improve parenting practices.

In this study, 6 one-hour WhatsApp support groups will be delivered to participating parents (maximum of 60 participants) by a trained m2m CHAMP facilitator. The facilitator will lead and monitor discussions and respond to any questions parents raise about technical troubleshooting and the content of the chatbot. At the end of the fifth WhatsApp group session, participants will be asked to select 2 peer leaders who will assume the responsibility of moderating the group after the sixth session.

**Figure 1:**
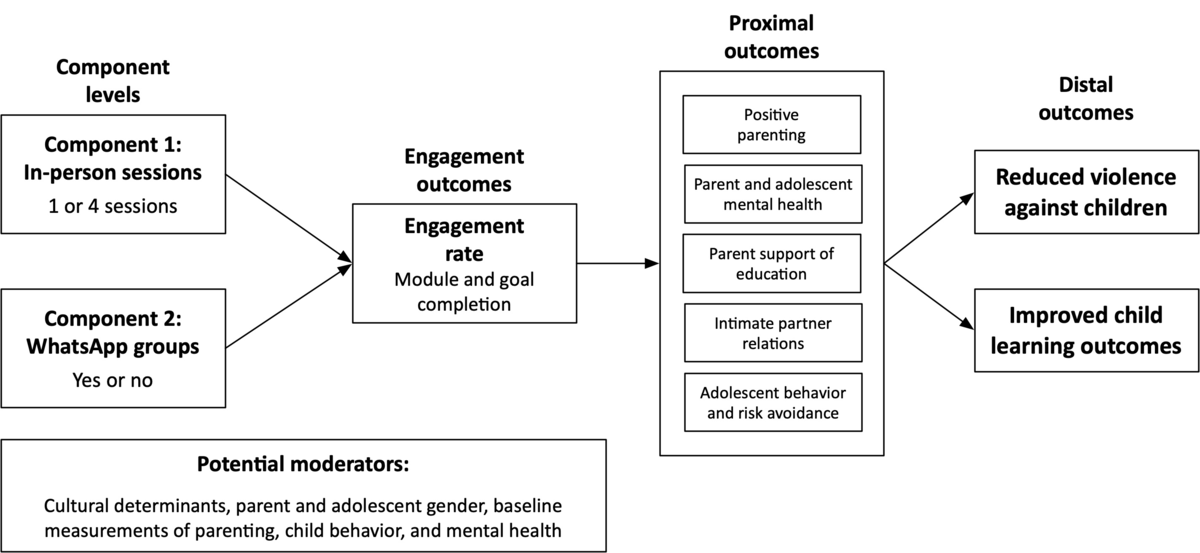
ParentText Optimisation Trial conceptual model.

As shown in Figure 1, the study components will contribute to increased engagement rates (eg, the number of modules and home practice activities completed within the parenting chatbot) among participants. Higher engagement rates will lead to more positive parenting practices and parent-teenager relationships, resulting in improved mental health and education outcomes for teenagers. Engagement with the chatbot will also contribute to improved intimate partner relationships and lead to positive mental health outcomes for parents. The sustainment of proximal outcomes will lead to a healthier environment within the household and contribute to reduced VAC and improved child learning outcomes.

### Training of Facilitators

m2m staff will be trained as program facilitators by certified trainers from CWBSA, a South African NGO with vast expertise in leading PLH program training. Training will occur over 2 days lasting a total of 12 hours. In total, 4 separate intervention manuals were developed to create a standardized delivery of the sessions based on condition allocation (ParentText+onboarding; ParentText+onboarding+WhatsApp support group; ParentText+onboarding+WhatsApp support group+3 in-person sessions; ParentText+onboarding+3 in-person sessions).

### Criteria for Discontinuing or Modifying Allocated Interventions

Adult and adolescent participants will have the right to decline any assessment or participation at any time during the study. Participants can opt out of receiving ParentText messages at any point by typing “EXIT” or simply by not responding to the messages. The decision to withdraw from the study will not affect a participant’s entitlement to other services or result in any penalty. Data collected through paper-based surveys and in-built assessments will be retained unless participants request otherwise.

### Strategies to Improve Adherence to the Intervention

Internet bundles (50 ZAR [approximately US $2.60] per person) will also be provided to support engagement with ParentText regardless of intervention condition allocation. Additional data bundles will also be provided to those allocated to participate in WhatsApp support groups.

### Relevant Concomitant Care Permitted or Prohibited During the Trial

Participants will not be prevented from receiving other care or services during the trial period.

### Provisions for Posttrial Care

Safeguarding procedures will be used to mitigate risks to participants. There is a possibility of participants disclosing violent practices toward their children, their partners, or themselves. The informed consent form indicates that some information regarding harmful behaviors will be disclosed without the participant’s consent if they pose a danger or harm to themselves or their family members. Participants at risk will be referred to local child welfare, health organizations, and other social services. Building on UNICEF’s safeguarding guidelines, ParentText has been designed to recognize high-risk keywords such as “trouble,” “SOS,” “ill,” “anxiety,” and “fire” in free-text fields. Participants can also type “HELP” to receive troubleshooting messages. The chatbot will respond to participants’ disclosure of dangerous situations automatically in an empathetic and empowering manner. It will also provide referral contacts localized in Mpumalanga to offer support to participants who are in danger of being harmed. All supporting services available for participants were identified by m2m and implementing teams following a meticulous mapping of local governmental and nongovernmental support systems. Finally, if respondents or their families are determined to have experienced significant harm because of participating in the study (ie, severe abuse, suicidality, IPV, or other potential psychological or physical injuries), we will cease further activities until these issues can be addressed adequately.

### Outcomes

Primary and secondary engagement outcomes will be collected through the course of the program implementation from August 21, 2023, to September 8, 2023.

#### Primary Outcomes: Engagement

The primary outcome related to user engagement is defined as the overall number of modules completed within the ParentText chatbot.

#### Secondary Outcomes

##### Engagement Outcomes

Secondary engagement outcomes will include the percentage of modules and goals completed. We will also examine the rate of self-reported completion of home practice activities based on the self-reported intention to do home practice activities. In total, 3 home practice activities will involve actual interaction with the chatbot, which will allow for testing whether users lie about completing home activities when prompted. Additional engagement outcomes will include the number of days users interact with the chatbot, number of triggered safeguarding messages, number of active and passive dropout from the chatbot, number of times users use the menu and option functions, number of times users access learning through play activities and type of activity (ie, calm, active, quick, or group), type of media selected for engagement (ie, text, audio, or video), and goal selected by users after completion of the first goal. For participants in all clusters, we will compute sum scores for each engagement outcome measure for the complete study period. High engagement rates will be defined as values standing at the highest quartile of the averaged engagement score, medium engagement rates will be defined as values at the second or third quartile, and low engagement rates will be defined as values at the lowest quartile.

##### Adult and Child Behavior Outcomes

Data collection of parent- and adolescent-reported mental health and behavior outcomes will occur at 2 time points: baseline and postintervention. Outcome measurements will be standardized to capture the occurrence of practices and behaviors over the “past month (last 30 days).”

1. *Child maltreatment (adult and adolescent report) * will be assessed using a subset of items from the physical abuse (2 items) and emotional abuse (2 items) subscales of the International Society for the Prevention of Child Abuse and Neglect Child Abuse Screening Tool–Trial Version [73].
2. *Positive parenting (adult and adolescent report)* will be assessed using a subset of items from the positive involvement (3 items), positive parenting (3 items), and parental supervision (3 items) subscales of the Alabama Parenting Questionnaire [74].
3. *Parent support of education (adult and adolescent report)* will be measured using 4 items assessing how often the parent engages the adolescent in behaviors that support learning [75].
4. *Mental health distress (adult and adolescent report)* will be measured using the 4-item Patient Health Questionnaire, which screens for anxiety and depression symptoms [76].
5. *Parenting stress (adult report only)* will be measured using the parental stressors subscale (6 items) of the Parental Stress Scale [77].
6. *Economic strengthening (adult report only)* will be measured using 1 item from the Financial Self-Efficacy Scale [78].
7. *Parent-child communication (adult report only)* will be measured using items adapted from the Parent-Child Communication Scale used in the Fast Track intervention study [79].
8. *Learning through play (adult report only)* will be assessed using a subset of items from the Alabama Parenting Questionnaire, the Parent-Child Communication Scale, and the items on parent support of education [74,75,80]. The selected scales will overarch the learning through play characteristics evidenced in the literature [57,81]. Parents will report the frequency of parent-child engagement [78].
9. *Intimate partner relationships (adult report only)* will be investigated by assessing gender-equitable behavior. Gender-equitable behavior will be measured using items adapted from the core questionnaire in the World Health Organization multicountry study on domestic violence [82] and from questionnaires used in previous violence prevention studies [83,84].
10. *Risk avoidance (adolescent report only)* will be assessed using 4 locally derived items on risk.
11. *Adolescent involvement in decision-making (adolescent report only)* will be assessed to investigate to what extent adolescents’ voices are heard within families regarding decisions affecting their lives. A total of 3 items adapted from questionnaires used in previous research on parent-child engagement in decision-making will be used to assess adolescent involvement in decisions related to their education, daily activities, and money expenditure in the household [85].
12. *Attitude toward punishment (adult and adolescent report)* will be assessed using a single item from the UNICEF Multiple Indicator Cluster Surveys 5 child discipline module [86].

ParentText also includes 1-item questions on parenting stress, parent-teenager interaction and communication, parental monitoring, teenage behavior, and intimate partner relationships that are embedded within the chatbot. These will be assessed both before and 1 week after the completion of each ParentText goal (total: 9 items).

##### Cultural Determinants of Engagement Outcomes

This study will investigate the cultural determinants associated with user engagement with ParentText through a parent-reported survey administered immediately after the in-person onboarding session. We will use the technology acceptance model 1 scale (16 items) to assess the following constructs: perceptions (ie, knowledge, attitudes, values, and beliefs affecting parents’ motivation for engaging with ParentText), enablers (ie, structural factors within society motivating parents’ engagement with ParentText, such as access to the internet), and nurturers (ie, the extent to which parents’ beliefs and attitudes are influenced and nurtured by their family, friends, and peers) [87]. Hedonic motivation (the perceived enjoyment of using the technology), habit (the extent to which an individual believes the use of a technology to be automatic), price value, and social influence were also included as additional variables to the technology acceptance model 1 due to their potential influence on the relationship between intention to use and engagement [88,89].

### Moderators and Covariates

Sociodemographic characteristics (adult and adolescent report) such as age, gender, literacy, disability, and family structure will be assessed at baseline to describe the sample population and investigate whether the intervention and components have differential effects across these subgroups.

Study assessments are summarized in Table 3.

**Table 3:** Summary of study measures.

			Measurement instrument		Study period						
					Baseline		Onboarding		Intervention		Posttest
**Primary engagement outcome**											
	Number of modules completed	—^a^						✓			
**Secondary engagement outcomes**									✓		
	Percentage of goals and modules completed	—						✓			
	Rate of self–home practice activity completion	—						✓			
	Total number of interactions	—						✓			
	Number of days of interaction	—						✓			
**Behavior outcomes**											
	Child maltreatment	ISPCAN^b^ Child Abuse Screening Tool–Trial Version (4 items)		✓						✓	
	Positive parenting	Alabama Parenting Questionnaire (9 items)		✓						✓	
	Parent support of education	4 items		✓						✓	
	Mental health distress	Patient Health Questionnaire (4 items)		✓						✓	
	Parenting stress	Parental Stress Scale (6 items)		✓						✓	
	Economic strengthening	Financial Self-Efficacy Scale (1 item)		✓						✓	
	Parent-child communication	Parent-Child Communication Scale (5 items)		✓						✓	
	Intimate partner relationships	WHO^c^ multi-country study on domestic violence (4 items)		✓						✓	
	Risk avoidance	6 items		✓						✓	
	Adolescent involvement in decision-making	3 items		✓						✓	
	Attitude toward punishment	Multiple Indicator Cluster Surveys (1 item)		✓						✓	
**Moderators and covariates**											
	Cultural determinants of engagement	Technology acceptance model 1 (16 items)				✓					
	Sociodemographic characteristics and family structure	For example, age, gender, literacy, and disability		✓							
**Cost outcomes**											
	Intervention costs	Staff time and expenditures related to the preparation and delivery of specific intervention components				✓		✓		✓	
Qualitative interviews and focus groups			—								✓

^a^The outcome indicated was not collected at the referred time point.

^b^ISPCAN: International Society for the Prevention of Child Abuse and Neglect.

^c^WHO: World Health Organization.

### Qualitative Assessment

Qualitative interviews with parents will investigate their overall experiences with the chatbot-led intervention, including the enrollment and interaction process. Questions will probe participants’ perceptions regarding the acceptability, usefulness, and cultural relevance of the chatbot, WhatsApp groups, and in-person sessions. Interviews will also explore the perceived impact of the intervention on parenting and family relationships.

### Participant Timeline

**Figure 2:**
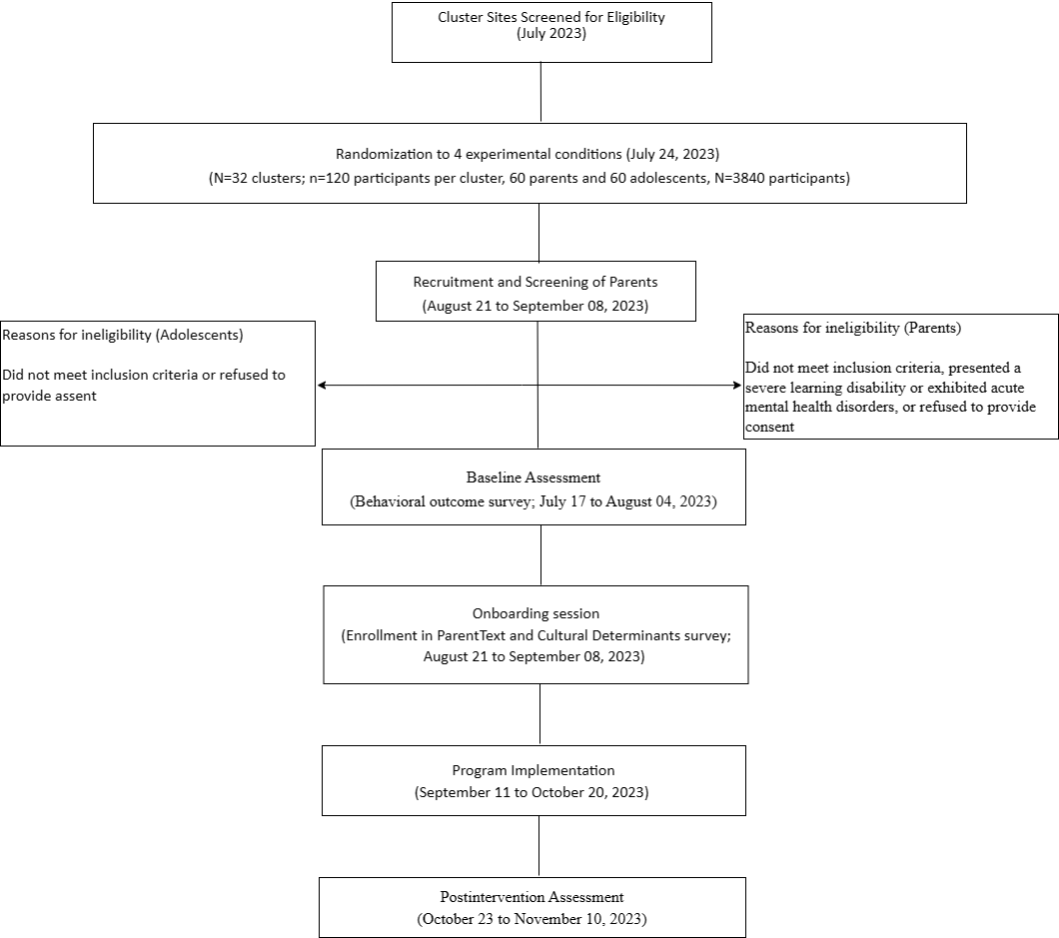
Study flow diagram.

The participant timeline is summarized in Figure 2. Sampling will be purposive and integrated into m2m CHAMP’s current recruitment strategy for the DREAMS project component. Recruitment will proceed until 60 parent-adolescent dyads per cluster are enrolled. Adult and adolescent participants will then be invited to an in-person session that will include (1) informed consent, (2) baseline assessment, (3) onboarding session, and (4) intention to use and cultural determinant survey (August 21, 2023, to September 8, 2023). Parents who complete the baseline assessment will receive a WhatsApp link to connect with ParentText and receive guidance on how to interact with the chatbot. The onboarding session will also include end-to-end testing to ensure that the chatbot functions correctly. Participants will be invited to interact with the chatbot for 5 minutes simulating a real-world interaction. They will then be asked to complete a paper-based assessment to collect their behavioral intention to use ParentText and cultural determinants. Depending on their assignment condition, parents will also be invited to join a facilitated WhatsApp support group or attend additional in-person sessions with their adolescent girls. The ParentText messages will be sent daily to parents via WhatsApp regardless of assignment condition. Data collectors will conduct posttest assessments approximately 6 weeks after the baseline (October 8-27, 2023). Follow-up assessments beyond the postintervention end point will not be conducted.

### Sample Size

The study has a fixed sample size by design with 32 clusters of equal size and equal treatment allocation across intervention arms. Sensitivity power analysis was conducted to estimate the minimum detectable effect size of the selected components on engagement and on behavior outcomes. Assuming a 2-sided test at the 10% level of significance varying the power (0.9 and 0.8) and the intracluster coefficient (0.02, 0.05, and 0.10), the minimum detectable effect ranges between a mean difference of 0.168 and 0.298 (80% power) and between a mean difference of 0.197 and 0.351 (90% power).

### Recruitment

Recruitment of participants will be based on purposive sampling relying entirely on 32 m2m CHAMP implementers and previous enrollment in the CHAMP program. CHAMP implementers will screen parents and their children to verify their eligibility for the trial. If required, recruitment will include peer-to-peer referrals in the community through respondent-driven sampling. Recruitment will proceed until 60 parent-adolescent dyads per cluster are enrolled. Participants will then be invited for a baseline assessment with trained data collectors where they will provide full written consent and complete the baseline assessment.

### Assignment of Interventions: Allocation

#### Sequence Generation

This study will adopt a cluster randomization procedure with allocation stratified by district using a parallel design. Cluster randomization will be stratified by facilitators and occur before the baseline assessments (12 clusters from Nkangala and 20 from Ehlanzeni). Random allocation will be conducted by the off-site research team in the United Kingdom using the Excel (Microsoft Corp) function “=Rand().” The in-country research team will assign clusters to 1 of the 4 experimental conditions (32 total clusters, 120 participants per cluster; 60 parents and 60 adolescent girls; N=3840 total participants). Using a 2 × 2 factorial design, this trial will randomly assign clusters so that parents receive the following engagement packages:

1. Engagement package 1: ParentText plus onboarding session for parents and adolescents led by an m2m facilitator
2. Engagement package 2: ParentText plus onboarding session for parents and adolescents and 6 moderated WhatsApp group sessions for parents only led by an m2m facilitator
3. Engagement package 3: ParentText plus onboarding session plus 3 in-person sessions for parents and adolescents and 6 moderated WhatsApp group sessions for parents only led by an m2m facilitator
4. Engagement package 4: ParentText plus onboarding session and 3 in-person sessions for parents and adolescents led by an m2m facilitator

#### Concealment Mechanism

Participants within a specific cluster and treatment condition will not be aware of the allocation of other clusters due to the geographical distance between clusters and lack of communication across groups.

#### Implementation

Participants will be recruited in blocks of 32 clusters linked by m2m. Households will be randomly selected from the assigned communities, and recruitment will proceed until 60 households are recruited in each block of 32 clusters.

### Assignment of Interventions: Blinding

#### Who Will Be Blinded

Due to the nature of the intervention, CHAMP facilitators, research assistants, and participants will be aware of condition allocation (based on cluster assignment). The range of conditions and allocation status of other enrolled clusters will be concealed from participants to reduce the risk of contamination. Trial statisticians conducting analyses on postintervention effects will be blinded to the assignment condition of clusters.

#### Procedure for Unblinding if Needed

This is not applicable for this study.

### Data Collection and Management

#### Plans for Assessment and Collection of Outcomes

This study will include quantitative and qualitative procedures for data collection. Data collected will consist of pretest-posttest parent and adolescent behavior questionnaires, parent cultural determinants of engagement, digitally tracked engagement data, and facilitator process data. All data will be collected using paper-and-pen forms. Individual semistructured interviews will be conducted to further explore participants’ engagement with ParentText and observed behavior outcomes.

#### Quantitative Data Collection

##### Baseline and Postintervention Surveys

Parent and adolescent behavior outcome surveys will be administered at baseline and the postintervention time point. Data collection will occur at the household level or at a meeting venue at the designated DREAMS schools. Data collectors will be 32 CHAMP implementers fluent in written English, isiZulu, or siSwati trained in ParentText delivery and study procedures such as interview techniques, research ethics, safety protocol, data management and security, informed consent, and adverse event report procedures. CWBSA and local researchers will conduct training and supervision of data collection. All measures, interview guides, workshop modules, and ParentText content will be translated from English into isiZulu and siSwati. Considering potential low literacy levels among some participants, data collection staff will read out the survey questions and response options, and participants will follow along. Facilitators will capture all data collected via Open Data Kit–based surveys (Get ODK Inc).

##### Cultural Determinants of Engagement Surveys

Cultural determinant outcome surveys will be collected at the ParentText onboarding session.

##### Chatbot Engagement Data

ParentText engagement data will be tracked digitally by the software developers, IDEMS International.

##### Engagement on Human Support Components

Facilitators will record in-person attendance and WhatsApp group discussion participation of parents enrolled in the cluster to which they were assigned.

##### Cost Data

Financial and economic costs associated with the resource inputs required to deliver the intervention and the intervention components will be considered in this study. Financial costs are defined as actual expenditures incurred on resource inputs, and economic costs are defined as the market value of all donated and subsidized resource inputs. Intervention costs include facilitator costs such as those associated with their training to deliver ParentText (captured as their time spent attending training sessions) and with preparing and delivering specific intervention components. Physical space used to deliver in-person sessions, as well as expenditures related to travel and supplies (such as internet data and cell phones), will be audited and valued. Local researchers and coordinators will collect resource use data *in* *real time* (ie*,* alongside the full study trial) via the completion of weekly Open Data Kit–based surveys. Costs related to the development of ParentText and content adaptation and translation will be included as a capital start-up cost. Because the cost analyses will be conducted from the provider perspective, costs incurred by participants (travel expenditure and opportunity costs) will be excluded. While the implementing organization’s program monitoring and evaluation costs will be included in cost estimates, broader research activity costs will be excluded.

#### Qualitative Data Collection

A subsample of parents will be purposively selected and invited via phone for in-person or over-the-phone individual semistructured interviews. Purposive selection will be based on gender and those with low, medium, and high levels of engagement (approximately 5 male and 5 female parents from different age groups). Engagement levels will be operationalized based on quartiles (ie, low engagement=lowest quartile; medium engagement=second and third quartile; high engagement=highest quartile). The subsample size will depend on the heterogeneity and availability of participants. To ensure that the interviews are standardized, interviewers will follow an interview guide developed by the research team. The interviews will be conducted by a trained qualitative researcher with the support of an interpreter. In addition, focus group discussions will be conducted with the implementing partner, m2m, to understand the challenges and opportunities during implementation.

#### Plans to Promote Participant Retention and Complete Follow-Up

To promote retention in the chatbot, participants will receive badges for each module completed, a trophy for each goal, and a certificate of completion at the end of the program. Participants attending the in-person sessions will be provided with refreshments at each session and receive a certificate of acknowledgment at the end of the fourth session.

### Data Management

This study will implement the following data management measures to ensure confidentiality, security, and safety. Per the US Agency for International Development implementation guidance, raw baseline and postintervention data collected will be captured to the Community-Based Intervention Monitoring System database, a South African community-based intervention monitoring system. Zoë-Life, a global training and consulting firm, will assist m2m with the deidentification of the data before they are transferred to the University of Oxford central server via an application programming interface. A Community-Based Intervention Monitoring System or system-generated ID will be assigned to uniquely identify each participant. ParentText engagement data will be automatically stored through end-to-end encryption to an IDEMS International server. During the data merging process, participants will be assigned a unique study code. The data on the University of Oxford central server will be managed by the University of Oxford Global Parenting Initiative data management team with technical support from the University of Oxford Department of Social Policy and Intervention IT team. Hard copies (eg, paper-and-pen questionnaires and participant attendance records of WhatsApp group and in-person sessions) will be stored in locked fireproof storage spaces and be permanently destroyed after being uploaded to a secure web-based data repository managed by the University of Oxford. Consent forms will be retained for at least 3 years after the study is published in compliance with the funders’ requirements.

### Confidentiality

Anonymized baseline and postintervention data sets will be stored in a password-protected server at the University of Oxford. Access will be controlled and only granted to members of the study team or partnered institutions that aided in the research project. Audio recordings of interviews and focus group discussions will be transcribed verbatim and stored in password-protected devices and will then be securely uploaded and backed up at the University of Oxford server. Once uploaded to the server, audio files will be deleted from the recording devices. After anonymizing and verifying the transcripts, audio recordings will be permanently deleted from data repositories. All Excel and Word (Microsoft Corp) files will be named following a standardized protocol, including the download date, to ease version control. Deidentified interview transcripts and data sets will only be shared by the Global Parenting Initiative data manager by granting access to specific files through the OneDrive for Business at the University of Oxford. Deidentified data sets will be stored securely for perpetuity and use per UK Data Archive guidance [90]. Raw baseline and postintervention data collected will be owned by the m2m South Africa office. Participants’ data will be stored by m2m in compliance with the South African Protection of Personal Information Act. IDEMS International will own the raw data collected through ParentText.

### Plans for Collection, Laboratory Evaluation, and Storage of Biological Specimens for Genetic or Molecular Analysis in This Trial or Future Use

This is not applicable for this study as no biological specimens for genetic or molecular analysis will be collected.

### Data Analysis

#### Statistical Methods for Primary and Secondary Outcomes

This study will examine the main effects of each component level on user engagement and behavior outcomes**.**

##### Engagement Outcomes

Baseline differences between groups will be described using proportions, means, and SDs. The main effect of each intervention component and interaction effects between components on primary and secondary engagement outcomes will be estimated using multilevel models (including mixed models if outcomes are continuous, Poisson models if outcomes are counts or count distributed, and logistic models if outcomes are binary). Separate models will be tested using effect coding [91,92]. Each model will specify 3 levels to account for the longitudinal and clustered nature of the data—repeated measures are nested within individuals, which are, in turn, nested within clusters. Level 1 will include a term for categorical time (pre- and posttest time points) and for the interaction between time and intervention status, level 2 will include terms for individual sociodemographics such as age (parent and adolescent) and gender (parent) and other individual-level covariates centered at the sample mean, and level 3 will include terms for the intervention components and the stratifying factor. Robust SEs will also be estimated to adjust for clustering. This study will report the direction and magnitude of β, incidence risk ratios, and odds ratios at a significance level of *P*<.10 with 90% CIs. Two-tailed tests will be conducted across all analyses.

##### Behavioral, Mental Health, and Educational Outcomes

The main effect of each intervention component and interaction effects between components on behavioral, mental health, and educational secondary outcomes will be estimated using the same multilevel models and specifying the same levels and structures as with the engagement outcomes. Within-group analyses will also be conducted to examine pretest-posttest differences across the sample accounting for clustering in analyses.

#### Analysis of Qualitative Data

Qualitative data will be analyzed using NVivo (QSR International) and Word through framework analysis [93]. Qualitative data analysis will be performed simultaneously with quantitative analysis to assess whether qualitative themes generalize to the entire population and expand the understanding of quantitative results. The analysis will include 5 stages: familiarization, identification of themes, indexing, charting and summarization, and interpretation and mapping. Researchers will begin by familiarizing themselves with the transcripts and identifying emerging themes. A code will then be assigned to each theme and subtheme at the indexing stage. After themes are indexed, they will be charted and summarized. Finally, interpretation and mapping will be used to understand the findings more deeply.

#### Cost-Effectiveness Analysis

This study will evaluate the efficiency and affordability that results from the expansion or contraction of the ParentText delivery package from the provider’s perspective. Program implementation costs and outcome data will be recorded and analyzed in Excel. The total costs of the intervention (setup and operating costs) for each intervention component and the average cost per participant enrolled (unit cost) will be estimated. The trial’s primary outcome relates to levels of user engagement (ie, the overall number of modules completed by participants), and this will be used to understand the cost-effectiveness of the intervention components that will maximize user engagement with ParentText. The estimated cost-to-engagement ratio for each of the intervention components will be calculated and compared to assess the incremental cost-effectiveness of each component. All costs will be adjusted to 2023 US dollars using the Consumer Price Index.

#### Analytical Strategy for Selecting the Most Optimal Intervention Package

**Figure 3:**
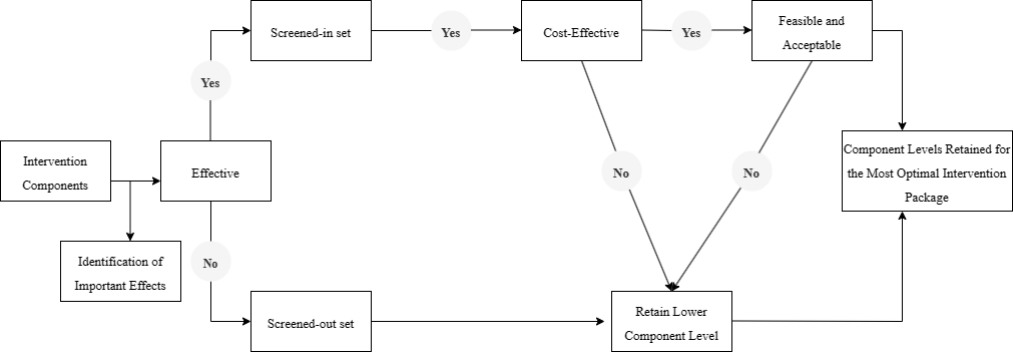
Analytical strategy for selecting the most optimal intervention package for evaluation in a subsequent randomized controlled trial.

We will use the decision-making framework presented by Collins [52] to select the most effective, cost-effective, and feasible components and component levels for inclusion in the optimized intervention before testing in a randomized controlled trial. As shown in Figure 3, the most optimal intervention package will be the one presenting higher effectiveness while considering resource and feasibility constraints. First, the main effects of each component on primary engagement outcomes will be identified and averaged across the other intervention components. Second, intervention components will be divided into a screened-in and screened-out set based on their positive effectiveness on participants’ engagement with ParentText. Intervention components with negative or no effect will be included in the screened-out set, and the lower level of that component will not be considered further. Third, intervention components in the screened-in set (ie, those showing positive effects and cost-effectiveness) will be considered for further evaluation. Results from qualitative interviews and focus group discussions conducted with the implementing partner will be used to further understand the feasibility, acceptability, effectiveness, and cost-effectiveness of the retained component levels and draw conclusions to develop the final optimized ParentText delivery package.

The posterior expected value approach will be included in the decision-making process to increase the robustness of our results. In previous research, the posterior expected value approach has modestly outperformed the component screening approach in accuracy, sensitivity, and specificity [94]. Estimates of the posterior expected value for each intervention component will be obtained using Markov chain Monte Carlo methods. We will prefer intervention components with higher Markov chain Monte Carlo posterior distribution of mean outcomes.

#### Interim Analyses

No interim analyses will be conducted.

#### Methods for Additional Analyses

Exploratory analysis will examine the effect of cultural determinants on parents’ behavioral intention to use ParentText. Pearson correlations will be used to determine the relationship between cultural determinants (perceived ease of use, perceived usefulness, hedonic motivation, habit, price value, and social influence) and behavioral intention toward ParentText use. Multiple regression analysis will be used to investigate the strength of the association between cultural determinants and behavioral intention and between behavioral intention and primary and secondary engagement outcomes. Predictive models will be used to investigate how behavioral intention predicts parents’ primary and secondary engagement outcomes.

#### Moderation Analyses

In addition, the study will use regression analysis and multilevel regression models to investigate the following: (1) which adult baseline characteristics moderate the effect of the intervention components on primary and secondary engagement outcomes and (2) which adult baseline characteristics moderate the effect of the intervention components on parent and adolescent behavioral, mental health, and educational outcomes.

#### Methods in Analysis to Handle Protocol Nonadherence and Any Statistical Methods to Handle Missing Data

Quantitative data analysis will be conducted on an intention-to-treat basis. Missingness will be inspected before analysis and addressed via multiple imputation techniques. Multiple imputation by chained equations will be favored to estimate item-level missing data when response patterns allow for estimation. Multiple imputation by chained equations allows more complex models involving moderations and multiple levels of mixed modeling to be accounted for.

### Plans to Give Access to the Full Protocol, Participant-Level Data, and Statistical Code

This research protocol and all components of this research, including but not limited to publications, fully anonymized data sets, data analysis plans, and code, will be available to the public at the Open Science Framework on an access-upon-request basis.

### Oversight and Monitoring

#### Composition of the Coordinating Center and Trial Steering Committee

A Trial Steering Committee (TSC) has been assembled and includes international experts with vast experience in experimental and implementation research and VAC prevention. The TSC will meet twice a year and at key study implementation time points (eg, after final data analyses) to ensure that the study is being conducted with rigor and respond to any arising issues associated with participant safety and study conduct.

#### Composition of the Data Monitoring Committee and its Role and Reporting Structure

The research team, including the principal investigators, research assistants and coordinators, and data managers, will monitor the full life cycle of data management, such as baseline and posttest collection, curation, storage, and analysis. Data quality checks will be conducted by creating a survey monitoring checklist to track errors (eg, wrong name in participant ID list, duplicated data entries, noneligible participants, and survey answers submitted incorrectly).

#### Adverse Event Reporting and Harms

Safeguarding strategies for each stage of this trial were created based on the universal principles of research ethics, respect, beneficence, and justice. The local research team will be trained on the safety protocol developed for this study. The local partner, m2m, also has a child safeguarding policy complying with the South African child welfare and protection legislation [95]. The policy includes guidelines for reporting suspected child abuse and exploitation. All m2m employers, partners, and other representatives have a duty to respond immediately (within 24 hours) to any allegations of child abuse and exploitation. They are trained to minimize risks and prevent harm to children.

Participants will be informed that ParentText is an entirely self-guided chatbot. However, building on UNICEF’s safeguarding guidelines, ParentText has been designed to recognize high-risk keywords such as “trouble,” “SOS,” “ill,” “anxiety,” and “fire” in free-text fields. The chatbot will respond to participants’ disclosure of dangerous situations automatically in an empathetic and empowering manner. It will also provide referral contacts localized in Mpumalanga to offer support to participants who are subject to being harmed. There is a possibility of participants’ disclosure of violent practices toward their children, their partners, or themselves. The informed consent form indicates that some information regarding harmful behaviors will be disclosed without the participant’s consent if they pose a danger or harm to themselves or their family members. Weekly supervision meetings with all qualitative project staff members will discuss issues that arise concerning harm to research participants and children. Finally, if we determine that respondents or their families have experienced significant harm because of participating in the study (ie, severe abuse, suicidality, IPV, or other potential psychological or physical injuries), we will cease further activities until these issues can be addressed adequately.

#### Frequency and Plans for Auditing Trial Conduct

Given that the intervention is being led by an external partner, trial conduct audits will be mainly processed by m2m. Deem it necessary, before any necessary actions or modifications affecting the implementation course of this intervention, m2m will communicate and seek advice from the TSC.

#### Plans for Communicating Important Protocol Amendments to Relevant Parties (eg, Trial Participants and Ethics Committees)

Any significant modifications to this protocol version 1.0 (August 7, 2023), including changes to objectives; design; recruitment; and data collection, storage, and analysis, will require a formal amendment. Modifications will be submitted for consideration and approval to the TSC and approved by the respective institutional research ethics committees. Minor administrative modifications not affecting the methodology described in this manuscript will be documented and reported to the TSC and ethics committees.

### Ethical Considerations

Ethics approval for this study was granted by the University of Cape Town Centre for Social Science Research Ethics Committee (reference: CSSR 2023/05), the University of Oxford Social Sciences and Humanities Interdivisional Research Ethics Committee (reference: R88177/RE001), and the Mpumalanga Department of Health and Department of Social Development (reference: R69569/RE003). Before the baseline assessments, participants will be asked to provide written informed consent by trained assessors under the supervision of the local research team. Participants assigned to facilitated WhatsApp groups will receive data bundles of 50 ZAR (approximately US $2.60) per person and a certificate of completion at the end of the sixth session. Study retention will be promoted by providing each parent and adolescent with 60 ZAR (approximately US $3.21) per person at each assessment point. Participants will be informed of any potential risks related to their participation in the trial and have the ability withdraw from the study without affecting any entitlement to other services or result in any penalty system. Deidentification of the data will be conducted before analysis and storage.

## Results

### Overview

The results of this study will be disseminated through publications in peer-reviewed journals, webinars, and conferences in South Africa and worldwide. Authorship of publications emerging from this study will adopt the recommended guidelines of the International Committee of Medical Journal Editors [96]. Brief reports containing relevant information and recommendations will also be created for stakeholders within NGOs, public health, and social care services in South Africa. Researchers will also provide verbal reports to local participants through local community meetings to help disseminate findings. Participant-identifiable details will be omitted in all research dissemination processes. The dissemination approach will provide the opportunity for early-career researchers and South African partners involved in this study to publish and present findings.

### Trial Status

Recruitment for the ParentText Optimisation Trial will begin on August 21, 2023, and is anticipated to continue until September 8, 2023. Baseline data collection will start on August 21, 2023, and is expected to be completed by September 8, 2023. This is the protocol version [1] of August 22, 2023.

## Discussion

### Expected Findings

Chatbot-led public health interventions offer a promising opportunity to scale up parenting programs in LMICs. However, it is crucial to understand the key pillars of user engagement to ensure their effective implementation. The ParentText Optimisation Trial is the first study to rigorously test engagement with a chatbot-led parenting intervention in an LMIC. This study brings a novel perspective to the research field of digital health interventions as it investigates the impact of engagement boosters associated with human support and the role of cultural determinants of health in designing a chatbot-led public health intervention. Moreover, the results that emerge from this trial will provide recommendations to enhance the development and delivery of interventions tailored for populations in LMICs. The identification of a context requiring lower resources and presenting higher acceptability and feasibility will enable the scale-up of digital public health interventions and support governments in improving populations’ health and addressing health inequalities.

## Appendix

Multimedia Appendix 1 Qualitative information sheet and consent forms for interviews.

Multimedia Appendix 2 Quantitative information sheets and consent for participation in surveys.

## Abbreviations

CHAMP: Children and Adolescents Are My Priority
CWBSA: Clowns Without Borders South Africa
DREAMS: Determined, Resilient, Empowered, AIDS-free, Mentored, and Safe
IDEMS: Innovations in Development, Education, and the Mathematical Sciences
IPV: intimate partner violence
LMIC: low- and middle-income country
m2m: mothers2mothers
mHealth: mobile health
NGO: nongovernmental organization
PLH: Parenting for Lifelong Health
TSC: Trial Steering Committee
UNICEF: United Nations Children’s Fund
VAC: violence against children

## Data Availability

No data sets or analysis were generated during the development of this manuscript as the research trial is still in the initial stage and no data have been collected at this point.
