# Supplementary material for: A Factorial Randomized Controlled Trial to Optimize User Engagement With a Chatbot-Led Parenting Intervention: Protocol for the ParentText Optimisation Trial
Source: JMIR Res Protoc. 2024 May 3;13:e52145. doi: 10.2196/52145 (PMC11102037; doi:10.2196/52145)
Supplement: Multimedia Appendix 1 [file resprot_v13i1e52145_app1.docx]

**Appendix 1: Qualitative Information Sheet and Consent Forms**

**Caregiver Consent for Qualitative Interviews**


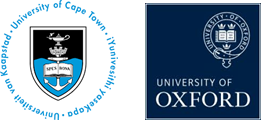

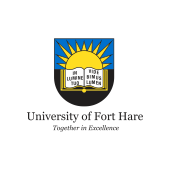


**CAREGIVER PARTICIPANT INFORMATION SHEET FOR PARTICIPATION IN INTERVIEWS**

Thank you so much for participating in the ParentText programme.You are now invited to take part in a one-on-one interview carried out by researchers from the Universities of Cape Town, Fort Hare, and Oxford, together with UNICEF South Africa, Clowns Without Borders South Africa, mothers2mothers (m2m), IDEMS International, and the South African Departments of Social Development, Health, and Education. This interview follows your participation in the ParentText programme and survey assessments, which you already provided consent for in August.

**Why is this study being conducted?**

We are doing this study to understand your experience with the ParentText programme. ParentText is a chatbot developed by Parenting for Lifelong Health (PLH) with the World Health Organization and UNICEF. We are interested in your experiences, thoughts, and opinions about the chatbot so that we understand how to improve the programme and help families like yours. We are also interested in changes in your family, relationships, and parenting before and after taking part in the programme.

**Why have I been invited to take part?**

You have been invited to take part in this study because you are a parent of a teen aged 10-17 and have been a part of the ParentText study. To participate in the interview, you will need to provide consent to confirm you are willing to take part.

**Do I have to take part?**

No, it is up to you to decide if you want to join. If you do not want to participate, you do not have to. You can stop taking part in the research at any time without anything bad happening. If you want to stop, please let one of the research team members know; you do not need to give any reason. We will stop recording and delete your personal information from the study.

**What will happen to me if I take part in the research?**

You can ask any questions that you have about the study. If you would like to take part, you will need to sign the consent form. We will contact you to arrange a time to ask you some questions about your thoughts and experiences on ParentText. We will also ask you some questions about any changes in your family, relationships, and parenting after the programme. This will either be done through interviews and/or a group interview with other families. We may also invite your teen to participate in the surveys and/or interviews or group discussions. Each interview will last between 1 hour and 1.5 hours.

You will have the right to skip questions you do not want to answer. There are also no right or wrong answers because your whole experience is important to us.

We will audio record the interview/group discussion to help us remember the discussion and write down what was said. We will give you a participant number, and you will be able to choose a name you want us to call you during the interview. The notes we make from the recordings will not include any information that will allow you or your teen to be identified. We will also collect information about you (such as your name, the age and gender of you and your children, your phone number and email address) to help us contact you during the study. We will delete this information at the end of the study.

If you report experiencing any problems and would want support from us, we will direct you to organisations that may be able to help. If we notice that you or your adolescent are at risk of serious harm, we may have to get help from external agencies.

**Are there any benefits to taking part?**

The information you provide will help us know whether ParentText makes a real difference for families in your community and other communities across the country.

**Are there any potential risks in taking part?**

We do not expect any risks in participating in this study. All information you give us will be kept confidential. If any questions make you feel uncomfortable, you don’t have to answer them and there will be no penalty against you for this decision. If you choose to be interviewed with your teen or in a group with other participants, we will define specific rules for participating and maintaining privacy, such as “anything a participant says should not be discussed outside the interview or focus group”. Although these rules will be set before starting the interview with your teen or group discussion with other participants, we cannot control what other participants may share outside the group.

If you become distressed or find anything that we talk about upsetting and would like to speak to someone afterwards, we can help to provide you with contact details where you can get support services. You can stop participating whenever you want to without giving a reason.

**What will happen to the information I provide?**

The personal information that we collect only includes what is necessary for the study. The information you share with us (e.g., your consent form, audio recording and written transcript of your interview) will be kept safe on a secure cloud server and backed up on servers at the Universities of Oxford, Fort Hare, and Cape Town.

We will keep your identifying details separately and only restricted study staff will have access. The rest of the information will be entered into another database, identified only by your study number, and we will only use this database to find the answers to our study questions. This means all information will remain confidential and private. Your information will not be kept longer than necessary. All the data will be stored for five years after the study, but the data that allows you to be identified (e.g., your name) will be destroyed at the end of the study. If you give us permission to record your interview, the recording will be deleted once we’ve written down what you said.

Study information may be reviewed by ethics committees and independent monitors to check that the study procedures were done correctly, and the information is correct. Your information will remain confidential unless we are required by law to release information.

When the study is finished, we will make information from the study available to be shared with other researchers. This will only be done after all the information which identifies people who took part has been removed, so the identities of the people who took part will remain confidential. You have the right to request access to your personal information at any time or request that we correct or destroy any information that you have provided before it is fully anonymised.

**What will happen to the results of the research?**

The results from the study will be presented to people working in the field of parenting programmes, to other researchers and to governments and other agencies in the form of presentations, publications in academic journals and policy briefs. In all these presentations and reports, it will not be possible to identify people who took part.

**Data protection**

The University of Oxford, Cape Town, and Fort Hare are responsible for ensuring the safe and proper use of any personal information you provide. We will only process data for research purposes. Research is a task that is performed in the public interest.

**Who has reviewed this study?**

This will receive approval from the University of Fort Hare, (**To insert the reference number)**, the University of Cape Town Department of Psychology’s Research Ethics Committee (**To insert the reference number)**, and the University of Oxford Ethics Committee **(To insert the reference number).**

**Funding**

This study is part of the Global Parenting Initiative, which is funded by the LEGO Foundation, Oak Foundation, the World Childhood Foundation (16191), The Human Safety Net, and the UK Research and Innovation Global Challenges Research Fund (ES/S008101/1). Funding for the implementation of ParentText is funded by USAID (72067418CA00026).

**Who do I contact if I have a question, a concern, or a complaint about the study?**

Please let us know if you have any questions about this interview/group discussion/survey before providing consent. Should you have any questions, concerns, or would like to complain about the study, please ask the research team who gave you this form or contact Dr Hlengiwe Gwebu at the University of Fort Hare. Her email address is [HGwebu@ufh.ac.za](mailto:HGwebu@ufh.ac.za).

If you have any further questions or concerns about your rights as a study participant, you can contact mothers2mothers or one of the following ethics committees:

| **Name** | **Telephone** | **Email** |
| --- | --- | --- |
| University of Cape Town | +27 21 650 3417 | Rosalind.Adams@uct.ac.za |
| University of Oxford | +44 1865616578 | Ethics@socsci.ox.ac.uk |
| University of Fort Hare | +27 043 704 7585 | aokeyo@ufh.ac.za |
| mothers2mothers | +27 66 536 6391 | Lindiwe.Mphahlele@m2m.org. |

Thank you!

*The study conforms to these data protection standards: GDPR (General Data Protection Regulation) in the UK and POPIA (Protection of Personal Information Act) in South Africa. Further information about your rights with respect to your personal data is available from https://compliance.admin.ox.ac.uk/individual-rights*

**Consent/Assent**

I have read or been read this information and understand it. I have had a chance to ask questions, and my questions have been answered. I understand that I can stop answering questions or going to the programme without penalty at any time by telling the facilitator. I understand who can see my information and how this information will be stored. I agree of my own free will to take part in the programme with my teen and to answer questions before and after the programme.

Central University Research Ethics Committee (CUREC) approval reference: xxxxx

|  | Please initial each box if you agree with the statement |
| --- | --- |
| I confirm that I have read and understand the information sheet version for the above research. I have had the opportunity to consider the information, ask questions and have had these answered satisfactorily. |  |
| I understand that my participation is voluntary and that I am free to withdraw at any point until 01/12/24, without giving any reason. |  |
| I understand who will have access to my personal data, how the data will be stored and what will happen to the data at the end of the project. |  |
| I understand that I will not be identifiable from any publications, reports, or presentations. |  |
| I consent to being audio recorded. |  |
| I understand how audio recordings will be used in research outputs. |  |
| Use of quotations: Please indicate your preference (select one option):  I do not wish to be quoted. or  I agree to the use of quotations in research outputs if I am not identifiable. |  |
| I give permission for you to contact me again to clarify information. |  |
| I understand how to raise a concern or make a complaint. |  |
| I agree to take part |  |
| I agree that my personal contact details can be retained in a secure database so that the researchers can contact me about future studies. | YES / NO |

______________________ dd / mm / yyyy ______________________

Name of participant Date Signature

______________________ dd / mm / yyyy ______________________

Name of person taking Date Signature

**Statement of Person Obtaining Informed Consent**

I have carefully explained to the person taking part in the study what he or she can expect from their participation. I confirm that this research subject speaks the language that was used to explain this research and is receiving an informed consent form in their primary language. This research subject has provided legally effective informed consent.

_____________________________________________ ____________

Signature of Person Obtaining Informed Consent Date

_____________________________________________

Printed Name of Person Obtaining Informed Consent

*The study conforms to these data protection standards: GDPR (General Data Protection Regulation) in the UK and POPIA (Protection of Personal Information Act) in South Africa. Further information about your rights with respect to your personal data is available from https://compliance.admin.ox.ac.uk/individual-rights*

**Consent for Implementing Partner Interviews/Focus Groups**


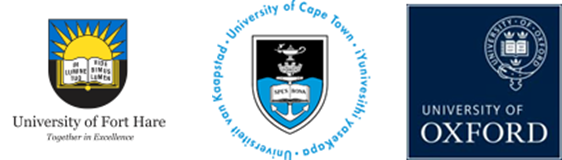


**PARTNER ORGANISATION INFORMATION SHEET FOR PARTICIPATION IN IMPLEMENTING PARTNER INTERVIEWS/FOCUS GROUPS**

Thank you so much for participating in the ParentText programme.

You are now invited to take part of a group discussion carried out by researchers from the Universities of Cape Town, Fort Hare, and Oxford, together with UNICEF South Africa, Clowns Without Borders South Africa (CWBSA), mothers2mothers (m2m), IDEMS International, and the South African Departments of Social Development, Health, and Education. This discussion follows your participation in the ParentText programme and survey assessments, which you already provided consent for in August.

**Why is this research being conducted?**

We are doing this study because we would like to test ParentText. ParentText is a chatbot developed by Parenting for Lifelong Health (PLH) with the World Health Organization and UNICEF. We are interested in your experiences, thoughts, and opinions about the chatbot so that we can better understand how to improve the lives of families with teens. We are also interested in changes in your family, relationships, and parenting before and after participating in the programme.

**Why have I been invited to take part?**

You have been invited to take part in this study because you work for a partner organisation who is assisting in the rollout and implementation of ParentText to parents of teens aged 10-17. We want to learn from your experience of implementing the app from your organisation with the aim of using your feedback to improve delivery in the future. To participate, you will need to provide consent to confirm you are willing to participate in the study.

**Do I have to take part?**

No, your participation is completely voluntary. This means that you do not have to participate. You should only take part if you want to volunteer. Even if you do agree to take part, you can stop taking part in the research at any time without any consequences or loss. If you want to withdraw, please let one of the research team members know; you do not need to give any reason, and there will be no penalty. Once you have let the research team know that you would like to withdraw, we will stop processing and delete your personal information from the study.

**What will happen to me if I take part in the research?**

If you agree to take part in this study, you will be given a chance to ask any questions that you have about the study and be asked to sign the consent form. With your permission, we will contact you to arrange a time to ask you some questions about your thoughts, experiences, and perceptions as someone who has implemented ParentText. We will also ask you some questions about your experiences and lessons learnt. This will either be done through interviews and/or focus group discussions. You will have the right to skip questions you do not want to answer. There are also no right or wrong answers because your whole experience is important to us. Each interview will last between 1 hour and 1.5 hours.

We will audio record the interview/group discussion to help us remember the discussion and write down what was said. We will give you a participant number, and you will be able to choose a name you want us to call you during the interview. The notes we make from the recordings will not include any information that will allow you to be identified. We will also collect information about you (such as your name, the age and gender, your phone number and email address) to help us contact you during the study. We will delete this information at the end of the study.

If you report experiencing any problems and would want support from us, we will direct you to organisations that may be able to help. If we notice that you or your adolescent are at risk of serious harm, we may have to get help from external agencies’.

**Are there any benefits to taking part?**

The information you provide will help us to ensure ParentText makes a real difference for families in your community and other communities across the country.

**Are there any potential risks in taking part?**

We do not expect any risks in participating in this study. All information you give us will be kept confidential. If any questions make you feel uncomfortable, you don’t have to answer them and there will be no penalty against you for this decision. If you choose to be interviewed with your teen or in a group with other participants, we will define specific rules for participation and confidentiality, such as “anything a participant says should not be discussed outside the interview or focus group”. Although these rules will be set before starting the interview with your teen or group discussion with other participants, we cannot control what other participants may share outside the group.

If you become distressed or find anything that we talk about upsetting and would like to speak to someone afterwards, we can help to provide you with contact details where you can get support services. Remember, you may at any point stop participating whenever you want to without giving a reason.

**What will happen to the information I provide?**

The personal information that we collect only includes what is necessary for the study. The information you share with us (e.g., your consent form, audio recording and written transcript of your interview) will be kept safe on a secure cloud server and backed up on servers at the University of Oxford and at the University of Cape Town.

We will keep your identifying details separately and only restricted study staff will have access. The rest of the information will be entered into another database, identified only by your study number, and we will only use this database to find the answers to our study questions. This means all information will remain confidential and private. Your information will not be kept longer than necessary. All the data will be stored for five years after the study, but the data that allows you to be identified (e.g., your name) will be destroyed at the end of the study. If you give us permission to record your interview, the recording will be deleted once we’ve written down what you said.

Study information may be reviewed by ethics committees and independent monitors to check that the study procedures were done correctly, and the information is correct. Your information will remain confidential unless we are required by law to release information.

When the study is finished, we will make information from the study available to be shared with other researchers. This will only be done after all the information which identifies people who took part has been removed, so the identities of the people who took part will remain confidential. You have the right to request access to your personal information at any time or request that we correct or destroy any information that you have provided before it is fully anonymised.

**What will happen to the results of the research?**

The results from the study will be presented to people working in the field of parenting programmes, to other researchers and to governments and other agencies in the form of presentations, publications in academic journals and policy briefs. In all these presentations and reports, it will not be possible to identify people who took part.

**Data protection**

The Universities of Oxford and Cape Town are responsible for ensuring the safe and proper use of any personal information you provide. We will only process data for research purposes. Research is a task that is performed in the public interest.

**Funding**

This study is part of the Global Parenting Initiative, which is funded by the LEGO Foundation, Oak Foundation, the World Childhood Foundation (16191), The Human Safety Net, and the UK Research and Innovation Global Challenges Research Fund (ES/S008101/1). Funding for the implementation of ParentText is funded by USAID (72067418CA00026).

**Who has reviewed this study?**

This will receive approval from the University of Fort Hare, (**To insert the reference number)**, the University of Cape Town Department of Psychology’s Research Ethics Committee (**To insert the reference number)**, and the University of Oxford Ethics Committee **(To insert the reference number).**

**Who do I contact if I have a question, a concern, or a complaint about the study?**

Please let us know if you have any questions about this interview/group discussion/survey before providing consent. Should you have any questions, concerns or would like to complain about the study, please ask the research team who gave you this form or contact Dr Hlengiwe Gwebu at the University of Fort Hare. Her email address is [HGwebu@ufh.ac.za](mailto:HGwebu@ufh.ac.za).

If you have any further questions or concerns about your rights as a study participant, you can contact mothers2mothers or one of the following ethics committees:

| **Name** | **Telephone** | **Email** |
| --- | --- | --- |
| University of Cape Town | +27 21 650 3417 | Rosalind.Adams@uct.ac.za |
| University of Oxford | +44 1865616578 | Ethics@socsci.ox.ac.uk |
| University of Fort Hare | +27 043 704 7585 | aokeyo@ufh.ac.za |
| mothers2mothers | +27 66 536 6391 | Lindiwe.Mphahlele@m2m.org. |

Thank you!

*The study conforms to these data protection standards: GDPR (General Data Protection Regulation) in the UK and POPIA (Protection of Personal Information Act) in South Africa. Further information about your rights with respect to your personal data is available from https://compliance.admin.ox.ac.uk/individual-rights*

**Consent/Assent**

I have read or been read this information and understand it. I have had a chance to ask questions, and my questions have been answered. I understand that I can stop answering questions or going to the programme without penalty at any time by telling the facilitator. I understand who can see my information and how this information will be stored. I agree of my own free will to take part in the programme with my teen and to answer questions before and after the programme.

Central University Research Ethics Committee (CUREC) approval reference: xxxxx

|  | Please initial each box if you agree with the statement |
| --- | --- |
| I confirm that I have read and understand the information sheet version for the above research. I have had the opportunity to consider the information, ask questions and have had these answered satisfactorily. |  |
| I understand that my participation is voluntary and that I am free to withdraw at any point until 01/12/24, without giving any reason. |  |
| I understand who will have access to my personal data, how the data will be stored and what will happen to the data at the end of the project. |  |
| I understand that I will not be identifiable from any publications, reports, or presentations. |  |
| I consent to being audio recorded. |  |
| I understand how audio recordings will be used in research outputs. |  |
| Use of quotations: Please indicate your preference (select one option):  I do not wish to be quoted. or  I agree to the use of quotations in research outputs if I am not identifiable. |  |
| I give permission for you to contact me again to clarify information. |  |
| I understand how to raise a concern or make a complaint. |  |
| I agree to take part |  |
| I agree that my personal contact details can be retained in a secure database so that the researchers can contact me about future studies. | YES / NO |

_____________________________________________ ____________

Signature of Person Taking Part in Study Date

_____________________________________________

Printed Name of Person Taking Part in Study

**Statement of Person Obtaining Informed Consent**

I have carefully explained to the person taking part in the study what he or she can expect from their participation. I confirm that this research subject speaks the language that was used to explain this research and is receiving an informed consent form in their primary language. This research subject has provided legally effective informed consent.

_______________________________________________________

Signature of Person Obtaining Informed Consent

Date ___________________________________________________

________________________________________________________

Printed Name of Person Obtaining Informed Consent

**Oral Consent Form**


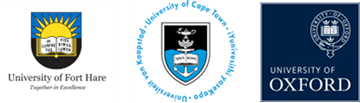


**PARENTTEXT ORAL CONSENT FORM**

**Oral consent script**

**Introduction**: Hello [again], my name is [x].Thank you so mcuh for participating in the ParentText programme.You are now invited to be part of a interview carried out by researchers from the Universities of Cape Town, Fort Hare, and Oxford, together with UNICEF South Africa, Clowns Without Borders South Africa, mothers2mothers (m2m), IDEMS International, and the South African Departments of Social Development, Health, and Education. This interview follows your participation in the ParentText programme and survey assessments, which you already provided consent for in August.

**Project details and aims**: We are doing this study because we would like to test ParentText. ParentText is a chatbot developed by Parenting for Lifelong Health (PLH) with the WHO and UNICEF. We are interested in your experiences, thoughts, and opinions about the chatbot so that we can better understand how to improve the lives of families with adolescents. We are also interested in changes in your family, relationships, and parenting before and after participating in the programme.

**Interviews/ focus group discussions:** With your permission, we will contact you to arrange a time to ask you some questions about your thoughts, experiences, and perceptions as someone who has participated in ParentText. [If applicable] We will also ask you some questions about any changes in your family, relationships, and parenting. This will either be done via an interview and or focus group discussion. You will have the right to skip questions you do not want to answer. There are also no right or wrong answers because your whole experience is important to us. The interview/ group discussion will last 60-90 minutes. With your permission, we will audio record the interview/group discussion to help us remember the discussion and write down what was said. We will give you a participant number, and you will be able to choose a name you want us to call you during the interview. The notes we make from the recordings will not include any information that will allow you or your adolescent to be identified. We will also collect information about you (such as your name, the age and gender of you and your children, your phone number and email address) to help us contact you during the study. We will delete this information at the end of the study.

If you report experiencing any problems and would want support from us, we will direct you to organisations that may be able to help. If we notice that you or your adolescent are at risk of serious harm, we may have to get help from external agencies’.

**Do I have to take part?**

No, your participation is completely voluntary. This means that you do not have to participate. You should only take part if you want to volunteer. Even if you do agree to take part, you can stop taking part in the research at any time without any consequences or loss. If you want to withdraw, please let one of the research team members know; you do not need to give any reason, and there will be no penalty. Once you have let the research team know that you would like to withdraw, we will stop processing and delete your personal information from the study.

**What will happen to me if I take part in the research?**

If you agree to take part in this study, you will be given a chance to ask any questions that you have about the study and be asked to sign the consent form. With your permission, we will contact you to arrange a time to ask you some questions about your thoughts, experiences, and perceptions as someone who has used ParentText. We will also ask you some questions about any changes in your family, relationships, and parenting. This will either be done through interviews and/or focus group discussions. You will have the right to skip questions you do not want to answer. There are also no right or wrong answers because your whole experience is important to us. With your permission, we may also invite your adolescent to participate in the surveys and/or interviews or group discussions.

You will be asked to participate an interview after the programme. You may participate in the interviews with your adolescent child, or separately from your adolescent child, whichever you prefer. We might do a group interview with other families. Each interview will last between 1 hour and 1.5 hours.

We will audio record the interview/group discussion to help us remember the discussion and write down what was said. We will give you a participant number, and you will be able to choose a name you want us to call you during the interview. The notes we make from the recordings will not include any information that will allow you or your adolescent to be identified. We will also collect information about you (such as your name, the age and gender of you and your children, your phone number and email address) to help us contact you during the study. We will delete this information at the end of the study.

**Are there any benefits to taking part?**

The information you provide will help us to ensure ParentText makes a real difference for families in your community and other communities across the country.

**Are there any potential risks in taking part?**

We do not expect any risks in participating in this study. All information you give us will be kept confidential. If any questions make you feel uncomfortable, you don’t have to answer them and there will be no penalty against you for this decision. If you choose to be interviewed with your adolescent or in a group with other participants, we will define specific rules for participation and confidentiality, such as “anything a participant says should not be discussed outside the interview or focus group”. Although these rules will be set before starting the interview with your teen or group discussion with other participants, we cannot control what other participants may share outside the group.

If you become distressed or find anything that we talk about upsetting and would like to speak to someone afterwards, we can help to provide you with contact details where you can get support services. Remember, you may at any point stop participating whenever you want to without giving a reason.

**What will happen to the information I provide?**

The personal information that we collect only includes what is necessary for the study. The information you share with us (e.g., your consent form, audio recording and written transcript of your interview) will be kept safe on a secure cloud server and backed up on servers at the University of Oxford and at the University of Cape Town.

We will keep your identifying details separately and only restricted study staff will have access. The rest of the information will be entered into another database, identified only by your study number, and we will only use this database to find the answers to our study questions. This means all information will remain confidential and private. Your information will not be kept longer than necessary. All the data will be stored for five years after the study, but the data that allows you to be identified (e.g., your name) will be destroyed at the end of the study. If you give us permission to record your interview, the recording will be deleted once we’ve written down what you said.

Study information may be reviewed by ethics committees and independent monitors to check that the study procedures were done correctly, and the information is correct. Your information will remain confidential unless we are required by law to release information.

When the study is finished, we will make information from the study available to be shared with other researchers. This will only be done after all the information which identifies people who took part has been removed, so the identities of the people who took part will remain confidential. You have the right to request access to your personal information at any time or request that we correct or destroy any information that you have provided before it is fully anonymised.

The Universities of Oxford of Cape Town are responsible for ensuring the safe and proper use of any personal information you provide. We will only process data for research purposes. Research is a task that is performed in the public interest.

**What will happen to the results of the research?**

The results from the study will be presented to people working in the field of parenting programmes, to other researchers and to governments and other agencies in the form of presentations, publications in academic journals and policy briefs. In all these presentations and reports, it will not be possible to identify people who took part.

**Who has reviewed this study?**

This will receive approval from the University of Fort Hare, (**To insert the reference number)**, the University of Cape Town Department of Psychology’s Research Ethics Committee (**To insert the reference number)**, and the University of Oxford Ethics Committee **(To insert the reference number).**

**Funding**

This study is part of the Global Parenting Initiative, which is funded by the LEGO Foundation, Oak Foundation, the World Childhood Foundation (16191), The Human Safety Net, and the UK Research and Innovation Global Challenges Research Fund (ES/S008101/1). Funding for the implementation of ParentText is funded by USAID (72067418CA00026).

**Who do I contact if I have a question, a concern, or a complaint about the study?**

Please let us know if you have any questions about this interview/group discussion/survey before providing consent. Should you have any questions, concerns or would like to complain about the study, please ask the research team who gave you this form or contact Dr Hlengiwe Gwebu at the University of Fort Hare. Her email address is [HGwebu@ufh.ac.za](mailto:HGwebu@ufh.ac.za).

If you have any further questions or concerns about your rights as a study participant, you can contact mothers2mothers or one of the following ethics committees:

| **Name** | **Telephone** | **Email** |
| --- | --- | --- |
| University of Cape Town | +27 21 650 3417 | Rosalind.Adams@uct.ac.za |
| University of Oxford | +44 1865616578 | Ethics@socsci.ox.ac.uk |
| University of Fort Hare | +27 043 704 7585 | aokeyo@ufh.ac.za |
| mothers2mothers | +27 66 536 6391 | Lindiwe.Mphahlele@m2m.org. |

Thank you!

**Consent/Assent**

I have been read this information and understand it. I have had a chance to ask questions, and my questions have been answered. I understand that I can stop answering questions or going to the programme without penalty at any time by telling the facilitator. I understand who can see my information and how this information will be stored. I agree of my own free will to take part in the programme with my teen and to answer questions before and after the programme.

Central University Research Ethics Committee (CUREC) approval reference: xxxxx

|  | Please initial each box if you agree with the statement |
| --- | --- |
| I confirm that I have read and understand the information sheet version for the above research. I have had the opportunity to consider the information, ask questions and have had these answered satisfactorily. |  |
| I understand that my participation is voluntary and that I am free to withdraw at any point until 01/12/24, without giving any reason. |  |
| I understand who will have access to my personal data, how the data will be stored and what will happen to the data at the end of the project. |  |
| I understand that I will not be identifiable from any publications, reports, or presentations. |  |
| I consent to being audio recorded. |  |
| I I understand how audio recordings will be used in research outputs. |  |
| Use of quotations: Please indicate your preference (select one option):  I do not wish to be quoted. or  I agree to the use of quotations in research outputs if I am not identifiable. |  |
| I give permission for you to contact me again to clarify information. |  |
| I understand how to raise a concern or make a complaint. |  |
| I agree to take part |  |
| I agree that my personal contact details can be retained in a secure database so that the researchers can contact me about future studies. | YES / NO |

_____________________________________________ ____________

Name of Participants Taking Part in Study Date

**Statement of Person Obtaining Informed Consent**

I have carefully explained to the person taking part in the study what he or she can expect from their participation. I confirm that this research subject speaks the language that was used to explain this research and is receiving an informed consent form in their primary language. This research subject has provided legally effective informed consent.

_____________________________________________________________

Signature of Person Obtaining Informed Consent

Date _______________________________________________

_______________________________________________________________

Printed Name of Person Obtaining Informed Consent
