# Supplementary material for: A Factorial Randomized Controlled Trial to Optimize User Engagement With a Chatbot-Led Parenting Intervention: Protocol for the ParentText Optimisation Trial
Source: JMIR Res Protoc. 2024 May 3;13:e52145. doi: 10.2196/52145 (PMC11102037; doi:10.2196/52145)
Supplement: Multimedia Appendix 2 [file resprot_v13i1e52145_app2.docx]

**Appendix 2: Quantitative Information Sheets and Consent Forms**

**Participant Information Sheet for Parents and/or Caregivers receiving ParentText and a Onboarding Session**

You are invited to be part of a study carried out by researchers from the Universities of Cape Town, Fort Hare, and Oxford, together with UNICEF South Africa, Clowns Without Borders South Africa, mothers2mothers (m2m), IDEMS International, and the South African Departments of Social Development, Health, and Education.

We are doing this study to learn about your experience with the ParentText chatbot, developed by Parenting for Lifelong Health (PLH) and UNICEF. The study is testing different ways of delivering the programme to support parents/caregivers using text messages, WhatsApp support groups, and in-person sessions. You have been randomly selected to receive text messages from ParentText plus an onboarding session with your teen to explain how the programme works. Before you decide whether you would like to take part, it is important for you to understand why the research is being done and what it will involve. An m2m facilitator will review this Participant Information Sheet with you.

If you have any questions about this study or do not understand something, please ask them. They are here to help you.

**Who can participate?**

To take part in the study, you need to be over 18 and have a teenage daughter between the ages of 10 and 17 years. You also need to provide consent to confirm you want to take part in the study.

**Do I have to participate?**

No, it's your choice to take part or not. If you don't want to participate, nothing bad will happen to you or your family. If you don't want to answer the survey, you can stop at any time and let the m2m facilitator know. You will still receive the ParentText messages even if you don't answer the questions. If you want to stop receiving messages, you can type "STOP MESSAGES" in the ParentText WhatsApp Chat.

**What will happen if I decide to participate?**

With your permission, you will be given a survey to answer. The survey will include questions about your parenting, relationship with your child and partner, well-being, and how your household manages money.You will be asked questions about your experience using ParentText. You can choose not to answer any question or withdraw from completing the survey at any time. These questions are important because they will help our research team understand how ParentText can help families.

Once the survey has been completed, you will be invited to join the ParentText programme. Once you join, you will receive daily ParentText messages via WhatsApp. If you want, you can interact with these messages. These messages have been designed to help you with parenting and your family’s well-being.

You will be interacting with the chatbot, but your teen can join you as you work through the ParentText programme, by reading the messages, listening and watching the audio and visual content, and by doing the home activities with you. If you report experiencing any problems and would like support, we can direct you to organisations that may be able to help. If we notice that you or your teen are at risk of serious harm, we may have to get help from external agencies.

You will also attend an in-person Onboarding session, where you will be taught how to use the ParentText chatbot and how the programme works. This session is designed to help you understand everything you need to know about the programme and our research. This session is also a great time for you to ask any further questions.

Your teen will also be invited to respond to a survey before and after the programme which they can choose to complete. Please note that she may choose to be asked questions without you present. You will not have access to any of your teen’s survey responses because all answers will be confidential. Your teen will be allowed to stop answering the survey at any point, without giving a reason.

**What will happen to the information I provide?**

All information you provide in the surveys will be completely private. Your name and any personal information about you and your family will NOT appear in any data, publications, or reports. Research data will be stored indefinitely and may be shared with other researchers in the future. The Universities of Cape Town, Fort Hare, and Oxford are responsible for ensuring the safe and proper use of any personal information you provide.

**What will happen to the results of the research?**

Your participation and any information you provide will help us learn about how to support families like yours. We plan to publish results in academic journals and policy briefs and present at conferences so that others can learn from this study.

**Who are the team members for this study?**

The principal investigators of this study are Dr Jamie Lachman (Universities of Oxford and Cape Town) and Dr Hlengiwe Gwebu (University of Fort Hare). The full research team includes Professor Frances Gardner, Dr Maria Ambrosio, Paula Zinser, Francisco Calderon, Dr Seema Vyas, Dr Inge Vallance (University of Oxford); Professor G.J. Melendez-Torres (University of Exeter); Dr David Stern, Chiara Facciola (IDEMS International); Anne Schley (m2m South Africa); and Laurie Markle (Parenting for Lifelong Health).

**Risks**

We do not expect any risks in taking part in this study. If you become upset when using the programme, you can find local referrals for additional support by typing “HelpMe” and selecting “Other Support”.

**Compensation**

Data bundles will be allocated to you at specific time points to support your engagement with ParentText.

**Funding**

This study is part of the Global Parenting Initiative, which is funded by the LEGO Foundation, Oak Foundation, the World Childhood Foundation (16191), The Human Safety Net, and the UK Research and Innovation Global Challenges Research Fund (ES/S008101/1). Funding for the implementation of ParentText is funded by USAID (72067418CA00026).

**Questions**

If you have any questions or concerns about your rights as a study participant, you can contact Dr Hlengiwe Gwebu at the University of Fort Hare Faculty of Health Science Department of Public Health via +27 (0) 67 307 6018 or [HGwebu@ufh.ac.za](mailto:HGwebu@ufh.ac.za).

If you have any further questions or concerns about your rights as a study participant, you can contact mothers2mothers or one of the following ethics committees:

| **Name** | **Telephone** | **Email** |
| --- | --- | --- |
| University of Cape Town | +27 21 650 3417 | [Rosalind.Adams@uct.ac.za](mailto:Rosalind.Adams@uct.ac.za) |
| University of Oxford | +44 1865616578 | [Ethics@socsci.ox.ac.uk](mailto:Ethics@socsci.ox.ac.uk) |
| University of Fort Hare | +27 043 704 7585 | [aokeyo@ufh.ac.za](mailto:aokeyo@ufh.ac.za) |
| mothers2mothers | +27 66 536 6391 | [Lindiwe.Mphahlele@m2m.org](mailto:Lindiwe.Mphahlele@m2m.org) |

**Consent/Assent**

I have read or been read this information and understand it. I have had a chance to ask questions, and my questions have been answered. I understand that I can stop answering questions or going to the programme without penalty at any time by telling the facilitator. I understand who can see my information and how this information will be stored. I agree of my own free will to take part in the programme with my teen and to answer questions before and after the programme.

**Central University Research Ethics Committee (CUREC) approval reference: xxxxx**

|  | **Please initial each box if you agree with the statement** |
| --- | --- |
| I confirm that I have read and understand the information sheet version for the above research. I have had the opportunity to consider the information, ask questions and have had these answered satisfactorily. |  |
| I understand that my participation is voluntary and that I am free to withdraw at any point until 01/12/2024, without giving any reason. |  |
| I understand who will have access to personal data provided, how the data will be stored and what will happen to the data at the end of the project. |  |
| I understand that I will not be identifiable from any publications or any reports or manuscripts that come from this study. |  |
| I give permission for you to contact me again to clarify information. |  |
| I understand how to raise a concern or make a complaint. |  |
| I agree to take part in the ParentText study. |  |
| I agree that my personal contact details can be retained in a secure database so that the researchers can contact me about future studies. | YES / NO |

______________________ dd / mm / yyyy ______________________

Name of participant Date Signature

______________________ dd / mm / yyyy ______________________

Name of person taking Date Signature
consent

**Participant Information Sheet for Parents and/or Caregivers receiving ParentText, a Onboarding Session and WhatsApp Group Support**

You are invited to be part of a study carried out by researchers from the Universities of Cape Town, Fort Hare, and Oxford, together with UNICEF South Africa, Clowns Without Borders South Africa, mothers2mothers (m2m), IDEMS International, and the South African Departments of Social Development, Health, and Education.

We are doing this study to learn about your experience with the ParentText chatbot, developed by Parenting for Lifelong Health (PLH) and UNICEF. The study is testing different ways of delivering a programme to support parents using text messages, WhatsApp groups, and in-person sessions. You have been randomly selected to receive text messages from ParentText, and participate in an onboarding session and a WhatsApp online support group.

Before you decide whether you would like to take part, it is important for you to understand why the research is being done and what it will involve. An m2m facilitator will review this Participant Information Sheet with you.

If you have any questions about the study or do not understand something, please ask them. They are here to help you.

**Who can participate?**

To take part in the study, you need to be over 18 and have a teenage daughter between the ages of 10 and 17 years. You also need to provide consent to confirm you want to take part in the study.

**Do I have to participate?**

No, it's your choice to take part or not. If you don't want to participate, nothing bad will happen to you or your family. If you don't want to answer the survey, you can stop at any time and let the m2m facilitator know. You will still receive the ParentText messages even if you don't answer the questions. If you want to stop receiving messages, you can type "STOP MESSAGES" in the ParentText WhatsApp Chat.

**What will happen if I decide to participate?**

With your permission, you will be given a survey to answer. The survey will include questions about your parenting, relationship with your child and partner, well-being, and how your household manages money.You will be asked questions about your experience using ParentText. You can choose to not answer any question or withdraw from completing the survey at any time. These questions are important because they will help our research team understand how ParentText can help families. Once the survey has been completed, you will be able to join the ParentText programme. Once you join, you will receive daily ParentText messages via WhatsApp. If you want, you can interact with these messages. These messages have been designed to help you with parenting and your family’s well-being.

You will be interacting with the chatbot, but your teen can join you as you work through the ParentText programme, by reading the messages, listening and watching the audio and visual content, and by doing the home activities with you. If you report experiencing any problems and would like support, we can direct you to organisations that may be able to help. If we notice that you or your teen are at risk of serious harm, we may have to get help from external agencies.

In addition to the ParentText programme, you will also participate in an in-person Onboarding session, where you will be taught how to use the ParentText chatbot and how the programme works. You will also be able to join a WhatsApp Support group. In this support group, you will be able to chat with your m2m facilitators and other caregivers who have joined the programme. You will be able to help and support each other with the programme.

Your teen will also be invited to respond to a survey before and after the programme which they can choose to complete. Please note that she may choose to be asked questions without you present. You will not have access to any of your teen’s survey responses because all answers will be confidential. Your teen will be allowed to stop answering the survey at any point, without giving a reason.

**What will happen to the information I provide?**

All information you provide in the surveys will be completely private. Your name and any personal information about you and your family will NOT appear in any data, publications, or reports. Research data will be stored indefinitely and may be shared with other researchers in the future. The Universities of Cape Town, Fort Hare, and Oxford are responsible for ensuring the safe and proper use of any personal information you provide.

**What will happen to the results of the research?**

Your participation and any information you provide will help us learn about how to better support families like yours. We plan to publish results in academic journals and policy briefs and present at conferences so that others can learn from this study.

**Who are the team members for this study?**

The principal investigators of this study are Dr Jamie Lachman (Universities of Oxford and Cape Town) and Dr Hlengiwe Gwebu (University of Fort Hare). The full research team includes Professor Frances Gardner, Dr Maria Ambrosio, Paula Zinser, Francisco Calderon, Dr Seema Vyas, Dr Inge Vallance (University of Oxford); Professor G.J. Melendez-Torres (University of Exeter); David Stern, Chiara Facciola (IDEMS International); Anne Schley (m2m South Africa); and Laurie Markle (Parenting for Lifelong Health).

**Risks**

We do not expect any risks in taking part in this study. If you become upset when using the programme, you can find local referrals for additional support by typing “HelpMe” and selecting “Other Support”.

**Compensation**

Data bundles will be allocated to you at specific time points to support your engagement with ParentText and participation in WhatsApp support groups.

**Funding**

This study is part of the Global Parenting Initiative, which is funded by the LEGO Foundation, Oak Foundation, the World Childhood Foundation (16191), The Human Safety Net, and the UK Research and Innovation Global Challenges Research Fund (ES/S008101/1). Funding for the implementation of ParentText is funded by USAID (72067418CA00026).

**Questions**

If you have any questions or concerns about your rights as a study participant, you can contact Dr Hlengiwe Gwebu at the University of Fort Hare Faculty of Health Science Department of Public Health via +27 (0) 67 307 6018 or [HGwebu@ufh.ac.za](mailto:HGwebu@ufh.ac.za).

If you have any further questions or concerns about your rights as a study participant, you can contact mothers2mothers or one of the following ethics committees:

| **Name** | **Telephone** | **Email** |
| --- | --- | --- |
| University of Cape Town | +27 21 650 3417 | [Rosalind.Adams@uct.ac.za](mailto:Rosalind.Adams@uct.ac.za) |
| University of Oxford | +44 1865616578 | [Ethics@socsci.ox.ac.uk](mailto:Ethics@socsci.ox.ac.uk) |
| University of Fort Hare | +27 043 704 7585 | [aokeyo@ufh.ac.za](mailto:aokeyo@ufh.ac.za) |
| mothers2mothers | +27 66 536 6391 | [Lindiwe.Mphahlele@m2m.org](mailto:Lindiwe.Mphahlele@m2m.org) |

**Consent/Assent**

I have read or been read this information and understand it. I have had a chance to ask questions, and my questions have been answered. I understand that I can stop answering questions or going to the programme without penalty at any time by telling the facilitator. I understand who can see my information and how this information will be stored. I agree of my own free will to take part in the programme with my teen and to answer questions before and after the programme.

**Central University Research Ethics Committee (CUREC) approval reference: xxxxx**

|  | **Please initial each box if you agree with the statement** |
| --- | --- |
| I confirm that I have read and understand the information sheet version for the above research. I have had the opportunity to consider the information, ask questions and have had these answered satisfactorily. |  |
| I understand that my participation is voluntary and that I am free to withdraw at any point until 01/12/2024, without giving any reason. |  |
| I understand who will have access to personal data provided, how the data will be stored and what will happen to the data at the end of the project. |  |
| I understand that I will not be identifiable from any publications or any reports or manuscripts that come from this study. |  |
| I give permission for you to contact me again to clarify information. |  |
| I understand how to raise a concern or make a complaint. |  |
| I agree to take part in the ParentText study. |  |
| I agree that my personal contact details can be retained in a secure database so that the researchers can contact me about future studies. | YES / NO |

______________________ dd / mm / yyyy ______________________

Name of participant Date Signature

______________________ dd / mm / yyyy ______________________

Name of person taking Date Signature
consent

**Participant Information Sheet for Parents and/or Caregivers receiving ParentText, a Onboarding Session and In-person Sessions**

You are invited to be part of a study carried out by researchers from the Universities of Cape Town, Fort Hare, and Oxford, together with UNICEF South Africa, Clowns Without Borders South Africa, mothers2mothers (m2m), IDEMS International, and the South African Departments of Social Development, Health, and Education.

We are doing this study to learn about your experience with the ParentText chatbot, developed by Parenting for Lifelong Health (PLH) with and UNICEF.  The study is testing different ways of delivering a programme to support parents using text messages, WhatsApp groups, and in-person sessions. You have been randomly selected to receive text messages from ParentText, an onboarding session, and in-person sessions with your teen.

Before you decide whether you would like to take part, it is important for you to understand why the research is being done and what it will involve. An m2m facilitator will review this Participant Information Sheet with you.

If you have any questions about this study or do not understand something, please ask them. They are here to help you.

**Who can participate?**

To take part in the study, you need to be over 18 and have a teenage daughter between the ages of 10 and 17 years. You also need to provide consent to confirm you want to take part in the study.

**Do I have to participate?**

No, it's your choice to take part or not. If you don't want to participate, nothing bad will happen to you or your family. If you don't want to answer the survey, you can stop at any time and let the m2m facilitator know. You will still receive the ParentText messages even if you don't answer the questions. If you want to stop receiving messages, you can type "STOP MESSAGES" in the ParentText WhatsApp Chat.

**What will happen if I decide to participate?**

With your permission, you will be given a survey to answer. The survey will include questions about your parenting, relationship with your child and partner, well-being, and how your household manages money. You will be asked questions about your experience using ParentText. You can choose to not answer any question or withdraw from completing the survey at any time. These questions are important because they will help our research team understand how ParentText can help families. Once the survey has been completed, you will be able to join the ParentText programme. Once you join, you will receive daily ParentText messages via WhatsApp. If you want, you can interact with these messages. These messages have been designed to help you with parenting and your family’s well-being.

You will be interacting with the chatbot, but your teen can join you as you work through the ParentText programme, by reading the messages, listening and watching the audio and visual content, and by doing the home activities with you. If you report experiencing any problems and would like support, we can direct you to organisations that may be able to help. If we notice that you or your teen are at risk of serious harm, we may have to get help from external agencies.

In addition to the ParentText programme you will also be participating in a in-person Onboarding session, where you will be taught how to use the ParentText chatbot, and three additional in-person sessions, where you will learn more about the programme.

Your teen will also be invited to respond to a survey before and after the programme which they can choose to complete. Please note that she may choose to be asked questions without you present. You will not have access to any of your teen’s survey responses because all answers will be confidential. Your teen will be allowed to stop answering the survey at any point, without giving a reason.

**What will happen to the information I provide?**

All information you provide in the surveys will be completely private. Your name and any personal information about you and your family will NOT appear in any data, publications, or reports. Research data will be stored indefinitely and may be shared with other researchers in the future. The Universities of Cape Town, Fort Hare, and Oxford are responsible for ensuring the safe and proper use of any personal information you provide.

**What will happen to the results of the research?**

Your participation and any information you provide will help us learn about how to better support families like yours. We plan to publish results in academic journals and policy briefs and present at conferences so that others can learn from this study.

**Who are the team members for this study?**

The principal investigators of this study are Dr Jamie Lachman (Universities of Oxford and Cape Town) and Dr Hlengiwe Gwebu (University of Fort Hare). The full research team includes Professor Frances Gardner, Dr Maria Ambrosio, Paula Zinser, Francisco Calderon, Dr Seema Vyas, Dr Inge Vallance (University of Oxford); Professor G.J. Melendez-Torres (University of Exeter); David Stern, Chiara Facciola (IDEMS International); Anne Schley (m2m South Africa); and Laurie Markle (Parenting for Lifelong Health).

**Risks**

We do not expect any risks in taking part in this study. If you become upset when using the programme, you can find local referrals for additional support by typing “HelpMe” and selecting “Other Support”.

**Compensation**

You will be provided with refreshments at each in-person session and receive a certificate of acknowledgement at the end of the fourth session. Data bundles will be allocated to you at specific time points to support your engagement with ParentText.

**Funding**

This study is part of the Global Parenting Initiative, which is funded by the LEGO Foundation, Oak Foundation, the World Childhood Foundation (16191), The Human Safety Net, and the UK Research and Innovation Global Challenges Research Fund (ES/S008101/1). Funding for the implementation of ParentText is funded by USAID (72067418CA00026).

**Questions**

If you have any questions or concerns about your rights as a study participant, you can contact Dr Hlengiwe Gwebu at the University of Fort Hare Faculty of Health Science Department of Public Health via +27 (0) 67 307 6018 or [HGwebu@ufh.ac.za](mailto:HGwebu@ufh.ac.za).If you have any further questions or concerns about your rights as a study participant, you can contact mothers2mothers or one of the following ethics committees:

| **Name** | **Telephone** | **Email** |
| --- | --- | --- |
| University of Cape Town | +27 21 650 3417 | [Rosalind.Adams@uct.ac.za](mailto:Rosalind.Adams@uct.ac.za) |
| University of Oxford | +44 1865616578 | [Ethics@socsci.ox.ac.uk](mailto:Ethics@socsci.ox.ac.uk) |
| University of Fort Hare | +27 043 704 7585 | [aokeyo@ufh.ac.za](mailto:aokeyo@ufh.ac.za) |
| mothers2mothers | +27 66 536 6391 | [Lindiwe.Mphahlele@m2m.org](mailto:Lindiwe.Mphahlele@m2m.org) |

**Consent/Assent**

I have read or been read this information and understand it. I have had a chance to ask questions, and my questions have been answered. I understand that I can stop answering questions or going to the programme without penalty at any time by telling the facilitator. I understand who can see my information and how this information will be stored. I agree of my own free will to take part in the programme with my teen and to answer questions before and after the programme.

**Central University Research Ethics Committee (CUREC) approval reference: xxxxx**

|  | **Please initial each box if you agree with the statement** |
| --- | --- |
| I confirm that I have read and understand the information sheet version for the above research. I have had the opportunity to consider the information, ask questions and have had these answered satisfactorily. |  |
| I understand that my participation is voluntary and that I am free to withdraw at any point until 01/12/2024, without giving any reason. |  |
| I understand who will have access to personal data provided, how the data will be stored and what will happen to the data at the end of the project. |  |
| I understand that I will not be identifiable from any publications or any reports or manuscripts that come from this study. |  |
| I give permission for you to contact me again to clarify information. |  |
| I understand how to raise a concern or make a complaint. |  |
| I agree to take part in the ParentText study. |  |
| I agree that my personal contact details can be retained in a secure database so that the researchers can contact me about future studies. | YES / NO |

______________________ dd / mm / yyyy ______________________

Name of participant Date Signature

______________________ dd / mm / yyyy ______________________

Name of person taking Date Signature
consent

**Participant Information Sheet for Parents and/or Caregivers receiving ParentText, an onboarding session, In person sessions and WhatsApp Group Support**

You are invited to be part of a study carried out by researchers from the Universities of Cape Town, Fort Hare and Oxford, together with UNICEF South Africa, Clowns Without Borders South Africa, mothers2mothers (m2m), IDEMS International, and the South African Departments of Social Development, Health, and Education.

We are doing this study to learn about your experience with the ParentText chatbot, developed by Parenting for Lifelong Health (PLH) and UNICEF.  The study is testing different ways of delivering a programme to support parents using text messages, WhatsApp groups, and in-person sessions. You have been randomly selected to receive text messages from ParentText, an onboarding session, 4 in-person sessions with your teen and to participate in a WhatsApp online support group.

Before you decide whether you would like to take part, it is important for you to understand why the research is being done and what it will involve. An m2m facilitator will review this Participant Information Sheet with you.

If you have any questions about this study or do not understand something, please ask them. They are here to help you.

**Who can participate?**

To take part in the study, you need to be over 18 and have a teenage daughter between the ages of 10 and 17 years. You also need to provide consent to confirm you want to take part in the study.

**Do I have to participate?**

No, it's your choice to take part or not. If you don't want to participate, nothing bad will happen to you or your family. If you don't want to answer the survey, you can stop at any time and let the m2m facilitator know. You will still receive the ParentText messages even if you don't answer the questions. If you want to stop receiving messages, you can type "STOP MESSAGES" in the ParentText WhatsApp Chat.

**What will happen if I decide to participate?**

With your permission, you will be given a survey to answer. The survey will include questions about your parenting, relationship with your child and partner, well-being, and how your household manages money. You will be asked questions about your experience using ParentText. You can choose to not answer any question or withdraw from completing the survey at any time. These questions are important because they will help our research team understand how ParentText can help families. Once the survey has been completed, you will be able to join the ParentText programme. Once you join, you will receive daily ParentText messages via WhatsApp. If you want, you can interact with these messages. These messages have been designed to help you with parenting and your family’s well-being.

You will be interacting with the chatbot, but your teen can join you as you work through the ParentText programme, by reading the messages, listening and watching the audio and visual content, and by doing the home activities with you. If you report experiencing any problems and would like support, we can direct you to organisations that may be able to help. If we notice that you or your teen are at risk of serious harm, we may have to get help from external agencies.

In addition to the ParentText programme, you will also participate in an in-person Onboarding session, where you will be taught how to use the ParentText chatbot and how the programme works. You will also receive 3 additional in-person sessions where you will learn more about the programme’s content. Lastly, you will be able to join a WhatsApp Support group. In this support group, you will chat with your m2m facilitators and other caregivers who have joined the programme. You will be able to help and support each other with the programme.

Your teen will also be invited to respond to a survey before and after the programme which they can choose to complete. Please note that she may choose to be asked questions without you present. You will not have access to any of your teen’s survey responses because all answers will be confidential. Your teen will be allowed to stop answering the survey at any point, without giving a reason.

**What will happen to the information I provide?**

All information you provide in the surveys will be completely private. Your name and any personal information about you and your family will NOT appear in any data, publications, or reports. Research data will be stored indefinitely and may be shared with other researchers in the future. The Universities of Cape Town, Fort Hare, and Oxford are responsible for ensuring the safe and proper use of any personal information you provide.

**What will happen to the results of the research?**

Your participation and any information you provide will help us learn about how to better support families like yours. We plan to publish results in academic journals and policy briefs and present at conferences so that others can learn from this study.

**Who are the team members for this study?**

The principal investigators of this study are Dr Jamie Lachman (Universities of Oxford and Cape Town) and Dr Hlengiwe Gwebu (University of Fort Hare). The full research team includes Professor Frances Gardner, Dr Maria Ambrosio, Paula Zinser, Francisco Calderon, Dr Seema Vyas, Dr Inge Vallance (University of Oxford); Professor G.J. Melendez-Torres (University of Exeter); David Stern, Chiara Facciola (IDEMS International); Anne Schley (m2m South Africa); and Laurie Markle (Parenting for Lifelong Health).

**Risks**

We do not expect any risks in taking part in this study. If you become upset when using the programme, you can find local referrals for additional support by typing “HelpMe” and selecting “Other Support”.

**Compensation**

You will be provided with refreshments at each in-person session and receive a certificate of acknowledgement at the end of the fourth session. Data bundles will be allocated to you at specific time points to support your engagement with ParentText and participation in WhatsApp support groups.

**Funding**

This study is part of the Global Parenting Initiative, which is funded by the LEGO Foundation, Oak Foundation, the World Childhood Foundation (16191), The Human Safety Net, and the UK Research and Innovation Global Challenges Research Fund (ES/S008101/1). Funding for the implementation of ParentText is funded by USAID (72067418CA00026).

**Questions**

If you have any questions or concerns about your rights as a study participant, you can contact Dr Hlengiwe Gwebu at the University of Fort Hare Faculty of Health Science Department of Public Health via +27 (0) 67 307 6018 or [HGwebu@ufh.ac.za](mailto:HGwebu@ufh.ac.za).

If you have any further questions or concerns about your rights as a study participant, you can contact mothers2mothers or one of the following ethics committees:

| **Name** | **Telephone** | **Email** |
| --- | --- | --- |
| University of Cape Town | +27 21 650 3417 | [Rosalind.Adams@uct.ac.za](mailto:Rosalind.Adams@uct.ac.za) |
| University of Oxford | +44 1865616578 | [Ethics@socsci.ox.ac.uk](mailto:Ethics@socsci.ox.ac.uk) |
| University of Fort Hare | +27 043 704 7585 | [aokeyo@ufh.ac.za](mailto:aokeyo@ufh.ac.za) |
| mothers2mothers | +27 66 536 6391 | [Lindiwe.Mphahlele@m2m.org](mailto:Lindiwe.Mphahlele@m2m.org) |

**Consent/Assent**

I have read or been read this information and understand it. I have had a chance to ask questions, and my questions have been answered. I understand that I can stop answering questions or going to the programme without penalty at any time by telling the facilitator. I understand who can see my information and how this information will be stored. I agree of my own free will to take part in the programme with my teen and to answer questions before and after the programme.

**Central University Research Ethics Committee (CUREC) approval reference: xxxxx**

|  | **Please initial each box if you agree with the statement** |
| --- | --- |
| I confirm that I have read and understand the information sheet version for the above research. I have had the opportunity to consider the information, ask questions and have had these answered satisfactorily. |  |
| I understand that my participation is voluntary and that I am free to withdraw at any point until 01/12/2024, without giving any reason. |  |
| I understand who will have access to personal data provided, how the data will be stored and what will happen to the data at the end of the project. |  |
| I understand that I will not be identifiable from any publications or any reports or manuscripts that come from this study. |  |
| I give permission for you to contact me again to clarify information. |  |
| I understand how to raise a concern or make a complaint. |  |
| I agree to take part in the ParentText study. |  |
| I agree that my personal contact details can be retained in a secure database so that the researchers can contact me about future studies. | YES / NO |

______________________ dd / mm / yyyy ______________________

Name of participant Date Signature

______________________ dd / mm / yyyy ______________________

Name of person taking Date Signature
consent

**Teen Quantitative Information Sheet and Assent Form**

Dear Teen,

We would like to invite you to be part of a research study focused on helping parents/caregivers improve their relationship with their teen girls. In this study we are working with the Universities of Cape Town, Fort Hare, and Oxford, UNICEF South Africa, Clowns Without Borders South Africa, mothers2mothers (m2m), IDEMS International, and the South African Departments of Social Development, Health, and Education.

We are doing this study to learn about your parent’s/caregiver’s experience using ParentText.

Before you decide whether you would like to participate, we need to tell you some things.  If you have any questions or do not understand something, please ask your m2m facilitator or your caregiver. Do not hesitate to talk to them, they are here to help you.

**What is ParentText?**

The programme is a text message-based programme (also called a chatbot) for parents/caregivers over 18 and their teens aged 10- to 17-years. It is designed to help build strong and positive relationships between parents/caregivers and their teens. **Do you have to take part?**

No. You can choose whether or not you want to take part in the study. If you feel uncomfortable participating in any of the activities related to the study or answering the survey you can choose to leave at any time without any negative consequences for you or your family.

**What will happen if I take part?**

A m2m CHAMP facilitator will ask you some general questions about yourself and your family before and after your parent/caregiver begins the ParentText programme. If your parent/caregiver starts the programme, you may be invited to attend one or up to four in-person sessions with them. You don't have to attend if you don't want to. Only your parent/caregiver will be messaging the ParentText chatbot, but you can read the messages, interact with the visual and audio content, and complete the home activities if you want to.

**Will anything about the research upset me?**

Some survey questions may be sensitive, especially those about your relationship with your parents/caregivers and your mental and physical well-being. If you report experiencing any problems and would like support, we can assist you in seeking appropriate support. Remember all your answers will be confidential. You can ask your caregiver to be present during the survey if you want to.

You do not have to answer any questions that you do not feel comfortable with. If you feel upset or want to talk to someone, the facilitatorr will explain some ways that you can get help. All this will be talked over with you first.

You will be allowed to leave the interview or any stage without giving a reason even if your parent/caregiver has not agreed. If we notice that you are at risk of serious harm, we may have to get help from external agencies’.

**What will happen to my information?**

We understand that providing personal information can be a little scary so we want to tell you that any data collected will be securely stored and only shared with other organisations and universities who want to understand more about parents and parenting programmes like ParentText. The information that they get will not include your name, address, phone number, or any other identifying information.All personal information will be deleted immediately after completing the study. So that others may learn from this study, we will share results in academic journals, at conferences, and in policy briefs for government and other agencies. Your name will not appear in any publications.

**What if there is a problem or something goes wrong?**

If you have any questions or concerns about the programme or questions, please talk to your parent/caregiver or contact your m2m facilitator If you have any questions or concerns about your rights as a study participant, you can contact Dr Hlengiwe Gwebu at the University of Fort Hare Faculty of Health Science, Department of Public Health via +27 (0) 67 307 6018 or [HGwebu@ufh.ac.za](mailto:HGwebu@ufh.ac.za). If you have any further questions or concerns about your rights as a study participant, you can contact Lindiwe Mphahlele at mothers2mothers in South Africa.

She can be contacted by e-mail at [Lindiwe.Mphahlele@m2m.org](mailto:Lindiwe.Mphahlele@m2m.org)

**Consent/Assent**

I have read or been read this information and understand it. I have had a chance to ask questions, and my questions have been answered. I understand that I can stop answering questions or going to the programme without any consequences at any time by telling the facilitator. I understand who can see my information and how this information will be stored. I agree of my own free will to take part in the programme with my parent/caregiver and to answer questions before and after the programme. My parent/caregiver, also gives me permission to participate.

**Central University Research Ethics Committee (CUREC) approval reference: xxxxx**

|  | **Please initial each box if you agree with the statement** |
| --- | --- |
| Somebody else explained this research project to me. |  |
| I understand what this project is about. |  |
| I have asked all the questions I wanted. |  |
| I have had my questions answered in a way I understand. |  |
| I understand it is OK to stop taking part at any time. |  |
| I am happy to take part in the ParentText Study. |  |
| I agree to be part of an interview in which my parents may be present. | YES / NO |
|  |  |

| Signature of teen giving assent  _______________________________________ |  |
| --- | --- |
| Printed name  _______________________________________ |  |
| Date:  _______________________________________ |  |

| Signature of caregiver giving consent  _______________________________________ | Signature of facilitator gaining consent  __________________________________________ |
| --- | --- |
| Printed name  _______________________________________ | Printed name  __________________________________________ |
| Date:  _______________________________________ | Place:  __________________________________________ |
